# Toward a Targeted Nutritional Strategy for Restoring PUFA Balance: Socio-Economic, Cultural and Ecologic Contexts, Biochemical Rationale, and a Conceptual Framework for Dietary Modulation

**DOI:** 10.3390/nu18101600

**Published:** 2026-05-18

**Authors:** Ulrich Suchner

**Affiliations:** Department of Anesthesiology and Surgical Intensive Care Medicine, Klinikum Darmstadt, 64283 Darmstadt, Germany; carpediemusu@outlook.de

**Keywords:** omega-6, omega-3, linoleic acid, seed oil, health, disease

## Abstract

This review outlines the health risks associated with excessive dietary intake of *n*-6 polyunsaturated fatty acids (PUFAs), particularly linoleic acid (C18:2*n*-6, LA), which is highly prevalent in the Western diet. It proposes a targeted nutritional strategy to reduce *n*-6 PUFA overconsumption and increase *n*-3 PUFA intake, aiming to restore a healthier fatty acid balance and counteract imbalance-induced pathogenetic consequences. The conceptual framework builds on the foundational insights of William E. M. Lands regarding PUFA-driven eicosanoid imbalance. It extends these principles by integrating contemporary models of impaired adipose tissue expandability, functional lipodystrophy, insulin resistance, and ectopic lipid deposition as central mechanisms of lipotoxicity and as unifying drivers of the modern organo-metabolic spectrum of non-communicable diseases. The proposed nutritional strategy combines dietary modifications—such as avoiding seed oils and processed foods as well as products from industrialized animal farming, while prioritizing fatty fish and/or algae-derived supplements—with lifestyle interventions and ongoing laboratory monitoring. This approach is designed to lower chronic disease risk and improve overall metabolic resilience. In addition, Western-diet-related socioeconomic issues and ecological burdens are addressed. The objective of this review is to evaluate the biochemical and clinical relevance of HUFA imbalance and to assess the potential of dietary modulation of *n*-6 and *n*-3 PUFAs as a strategy to restore metabolic homeostasis. However, further research is required to corroborate the available findings before broader implementation of the proposed strategy can be recommended.

## 1. Introduction

There are compelling reasons to assume that lowering dietary linoleic acid (C18:2*n*-6, LA) intake in industrialized and several emerging countries could yield clinically relevant metabolic benefits [1]. This concept was first articulated by Bill Lands in the 1980s [2] and further refined in the early 2000s [3,4,5]. We recently revisited this framework from a contemporary perspective, emphasizing its continued relevance for current dietary patterns [6]. The rationale appears particularly applicable in settings characterized by increased availability of bioactive derivatives of *n*-6 highly unsaturated fatty acids (HUFAs) and simultaneously reduced availability of bioactive *n*-3 HUFA derivatives, both belonging to the broader class of polyunsaturated fatty acids (PUFAs) [7]. Moreover, Lands’ work demonstrated that—given the limited intake of counterbalancing *n*-3 PUFA-rich foods in Western dietary patterns—the *n*-6 PUFA LA and its metabolites exhibit a narrow “therapeutic window,” providing physiological benefits only within a restricted intake range, whereas even modest upward deviations can disproportionately shift HUFA balance and downstream inflammatory signaling [6].

In parallel with the rise in the endogenous *n*-6/*n*-3 PUFA ratio, the incidence of cancer and other non-communicable diseases—including hypertension, atherosclerosis, cardiovascular disease, diabetes mellitus, and metabolic syndrome—has increased. Mechanistic models and selected epidemiological studies suggest that a high *n*-6/*n*-3 fatty acid ratio may promote pro-inflammatory and cardiometabolically unfavorable profiles [8,9,10]. Experimental evidence further indicates that *n*-6–derived oxylipins and reduced availability of *n*-3–derived specialized pro-resolving mediators can shift inflammatory and metabolic pathways toward dysfunction [10]. Observational biomarker studies likewise associate higher *n*-6/*n*-3 ratios with elevated inflammatory markers and adverse cardiometabolic traits [9]. Signals in the same direction have also been reported in individual randomized controlled trials (RCTs), as summarized in a recent RCT-based systematic review, although these findings require cautious interpretation due to heterogeneous study designs and generally limited evidence quality [11].

Nevertheless, taken together, these converging lines of evidence raise the question of why they have not yet been translated into a nutritional strategy that explicitly targets a reduction in *n*-6 PUFA intake. Current approaches have focused almost exclusively on increasing the consumption of *n*-3 PUFA-rich foods, a strategy with only limited potential for success [6]. Our considerations suggest that enhancing *n*-3 PUFA intake is likely to be more effective when implemented alongside a concurrent reduction in dietary *n*-6 PUFAs. This combined approach provides a rationale for an innovative nutritional strategy aimed at lowering LA availability in selected individuals or patients—particularly in those with elevated tissue levels of *n*-6 PUFAs or HUFAs—either for preventive or potentially therapeutic purposes.

However, the analyses presented in this manuscript are not intended as normative dietary guidance or as value judgments on specific food categories or agricultural systems. Rather, the discussion aims to synthesize biochemical, epidemiological, and ecological evidence relevant to PUFA exposure and HUFA balance. Any references to dietary patterns or oil sources should therefore be interpreted within this analytical framework and not as categorical recommendations.

The purpose of this review is to examine the biochemical and clinical relevance of HUFA imbalance, to evaluate the evidence supporting dietary modulation of *n*-6 and *n*-3 PUFAs as a potential strategy to restore metabolic homeostasis, and to contextualize these considerations within broader socio-economic and ecological trends in Western dietary patterns.

The present article is designed as a narrative review. It builds on a previous publication that revisited and synthesized Bill Lands’ hypotheses [6], which have been in the public domain for more than four decades but have received comparatively little scientific attention. Whereas the earlier article provided a historical and conceptual reconstruction of Lands’ framework, the current manuscript extends this work by examining its relevance in light of recent advances in organo-metabolic pathophysiology, by exploring whether a dietary strategy aimed at normalizing HUFA imbalance can be formulated and evaluated, and by considering the broader socio-economic, cultural, and ecological implications of the global expansion of oilseed production and consumption.

A formal systematic review or meta-analysis was not conducted, as such an approach would require methodological prerequisites that most available studies do not meet—particularly the concurrent assessment of both PUFA families, HUFA profiling, and the modeling of their competitive and non-linear interactions. The literature included in this manuscript was therefore selected based on conceptual relevance to Lands’ framework and its extensions, with the aim of illustrating areas of agreement, controversy, and uncertainty across mechanistic, observational, and interventional evidence. The selection process followed the logic of narrative synthesis rather than predefined inclusion or exclusion criteria.

The manuscript was edited with the assistance of AI-based language tools (Microsoft Copilot, 2025 version) to improve clarity and readability. All scientific content, interpretations, and conclusions were developed solely by the author.

## 2. Background Overview of the Proposed Nutrition Strategy

The draft nutrition strategy proposed here is based on the following background information:

### 2.1. Linoleic Acid (LA) Has Become Substantially More Abundant in Modern Diets Since the Mid-20th Century

Linoleic acid (C18:2*n*-6, LA) and alpha-linolenic acid (C18:3*n*-3, ALA) are essential fatty acids that cannot be synthesized endogenously; however, the central issue addressed in this review is not their essentiality but the disproportionately high LA intake characteristic of modern dietary patterns.

Industrial processing has made seeds a major component of the global food system [12,13], and the resulting seed oils (e.g., soybean, maize, rapeseed/canola, sunflower, safflower) are now widely used in food manufacturing, household cooking, and even in enteral and parenteral nutrition products. Their use as livestock feed further increases the *n*-6 PUFA content of meat and dairy products compared with historical patterns. Consequently, populations consuming a Western dietary pattern are exposed to markedly higher levels of *n*-6 PUFAs than in the past [13].

Historical analyses indicate that LA contributed only about 2–3% of total energy intake in the early 20th century, before the widespread adoption of industrially produced seed oils. Over subsequent decades, shifts from animal fats toward soybean, maize, sunflower, and other vegetable oils—documented in FAO/WHO reports [14]—led to a substantial rise in LA availability within the food supply. Contemporary intake data from NHANES confirm that LA now contributes approximately 5–7% of energy in U.S. adults, reflecting the pervasive use of seed oils in processed foods, household cooking, and clinical nutrition products [15]. Similar trends have been described in parts of Europe, where increasing reliance on sunflower and soybean oils has elevated population-level LA exposure [16]. Together, these findings demonstrate a clear and sustained shift toward higher dietary *n*-6 PUFA intake in Western populations, driven both by direct consumption of seed oils and by indirect enrichment of animal-derived foods through modern feeding practices.

Although this trend is well established, quantitative comparisons of LA intake across populations remain surprisingly difficult due to a lack of good data. Most large-scale surveys report absolute LA intake (g/day) rather than percentage of energy (%En LA), and traditional populations are rarely characterized with modern nutrient analytics. As a result, cross-population comparisons require a combination of direct intake data, calculated values, and inferences from dietary structure. While approximate, such reconstructions currently provide the only coherent overview of global variation in LA exposure.

To address this gap, we synthesized available intake data, converting reported LA values (g/day) to percentage of energy (%En LA) whenever possible and using biomarker evidence (erythrocyte/plasma LA) as contextual support. Because directly reported %En LA values are scarce, all calculations followed a standardized approach in which LA intake (g/day) was combined with typical energy intake (kcal/day) using the conventional conversion of 9 kcal/g:

$$\%En_{\mathrm{LA}}\approx \;\frac{\mathrm{LA}\;(g/\mathrm{day})\times 9}{\mathrm{total}\;\mathrm{energy}\;(\mathrm{kcal}/\mathrm{day})}\times 100.$$

where quantitative intake data were unavailable, %En_LA_ was inferred from characteristic dietary patterns (e.g., reliance on seed oils, coconut, animal fats, or marine fats). Biomarker data were used as integrative indicators of longer-term exposure but not as direct substitutes for %En. All resulting values should therefore be interpreted as order-of-magnitude estimates, with explicit indication of whether they are directly reported, calculated, or inferred.

Approximate values for %En LA across selected populations are summarized in Table 1. Western industrialized populations typically fall within ~4–7%En LA, reflecting high consumption of LA-rich seed oils. Mediterranean and Japanese dietary patterns show moderate LA intake (~3–5%En), whereas traditional horticulturalist populations (e.g., Kitava) exhibit very low LA intake (<2%En) due to minimal seed-oil consumption and reliance on coconut, tubers, fruits, and local animal foods.

Biomarker data support the direction of these differences but do not provide direct %En values. To enhance transparency, all values in Table 1 are explicitly categorized as measured (directly reported intake data), calculated (derived from reported LA intake and typical energy intake), or inferred (estimated from characteristic dietary patterns supported by biomarker profiles). These categories reflect the heterogeneous nature of available data and are intended to guide interpretation of the table as an order-of-magnitude comparison rather than as a set of precise quantitative measurements.

The variation in approximate %En LA across populations carries clinical relevance when viewed through the lens of Lands’ concept of a “therapeutic window” for *n*-6 to *n*-3 PUFA balance. Lands proposed that eicosanoid signaling remains physiologically stable only within a relatively narrow range of substrate competition between AA-derived and EPA/DHA-derived mediators. Populations with very low LA intake, such as traditional horticulturalist groups, likely operate near the lower boundary of this window, where *n*-3 PUFA–derived pathways predominate and inflammatory tone remains low. In contrast, Western populations consuming ~5–7%En LA appear to exceed the upper boundary, shifting desaturase competition and downstream lipid mediator synthesis toward *n*-6–derived, pro-inflammatory pathways. Dietary patterns such as the traditional Mediterranean or Japanese diets, which fall into an intermediate range (~3–5%En LA), may lie closer to Lands’ proposed equilibrium point and could help explain their favorable cardiometabolic profiles. From a clinical and nutritional strategy perspective, these observations suggest that moderating LA intake toward this intermediate range—while ensuring adequate *n*-3 PUFA availability—may help restore a more favorable eicosanoid balance and support long-term metabolic and inflammatory homeostasis. This interpretation aligns with Lands’ kinetic model of *n*-6/*n*-3 substrate competition, which describes a narrow physiological range in which eicosanoid signaling remains stable and predicts a shift toward *n*-6–derived mediators once LA intake exceeds this range [22].

### 2.2. Global Oilseed Production in Recent Decades Causes Major Socio-Economic, Cultural and Ecological Burdens

A rigorous quantitative assessment of the global economic, cultural, and ecological consequences of industrial oilseed cultivation and processing requires systematic examination of the underlying primary data. Global production volumes and cultivation areas for important oilseeds and vegetable oils are shown in Table 2. Today, global oilseed production has expanded to approximately 675–680 million metric tons per year—placing its mass output in the same order of magnitude as global wheat production and global rice production together when measured at the paddy stage [23,24,25]. These crops now occupy more than 300 million hectares of land, an area comparable to the landmass of India and roughly one fifth of all global agricultural land [23,24,25]. This expansion is driven by rising demand for vegetable oils, protein-rich feed ingredients, and biofuel feedstocks, concentrating agricultural growth in ecologically sensitive regions across the Americas, Europe, and Southeast Asia [26,27,28].

For the 2023/24 harvest year, global soybean production reached an all-time high of ~405 million tons, up from ~220 million tons in 2005/06 [29]. Brazil and the United States dominate global production and account for more than 85% of global soybean exports, while China remains the largest importer by a wide margin [29]. Within Europe, Italy, France, and Serbia are the most important producers, whereas Germany and other EU countries increasingly rely on imports for food and feed.

The global vegetable oil market itself was estimated at 199 million tons in 2020 and in the meantime has further grown, reaching ~234 million tons in 2025/26, with palm oil (~80 million tons), soybean oil (~70 million tons), rapeseed oil (~32 million tons), and sunflower oil (~23 million tons) dominating global output [23,30,31]. The fatty-acid composition of these oils is highly relevant for human nutrition: linoleic acid (LA) constitutes 71% of total fatty acids in sunflower oil, 56% in cottonseed oil, 56% in soybean oil, and 31% in peanut oil, whereas palm kernel oil contains only ~3% LA [32]. Oil palm plantations alone cover ~30 million hectares, while soybean, rapeseed, and sunflower cultivation occupy more than 215 million hectares combined [24,33]. Although these oils underpin global food, feed, and industrial value chains, their production is tightly linked to land-use change, deforestation, and biodiversity loss [34,35,36].

Soybean oil is the second-largest vegetable oil globally. Its land footprint is inseparable from the broader soybean complex, which spans ~144 million hectares worldwide [12,23,25]. The overwhelming majority of soybeans—75–80%—are processed into meal for industrial livestock systems, supplying poultry, pork, cattle, and aquaculture sectors [24]. More than 300 million tons of soybeans each year are therefore directed to animal feed. This has nutritional consequences: the high *n*-6 PUFA content of soybean-based feed is incorporated into the cell membranes and adipose tissue of livestock, thereby increasing the *n*-6 PUFA burden in meat, dairy, and farmed fish consumed by humans. Soybean isolates are also widely used in aquaculture, including for farmed salmon. Without massive soybean imports, industrial livestock farming in its current form would not be possible.

Globally, pork and poultry are the dominant monogastric meat sources, with pork still exceeding 120 million tons and poultry surpassing 135 million tons annually [37]. Their cereal–soy–oil feeding systems link global oilseed agriculture directly to human PUFA exposure, because monogastrics incorporate dietary fatty acids with minimal modification. Not only soybean oil and other seed oils but also cereals contribute substantial LA: maize contains ~55–60% LA, wheat ~50–55%, and barley ~45–50% of total fatty acids [38], making cereal-based rations significant *n*-6 PUFA providers even before added oils are included. Consequently, pork and poultry production acts as a major upstream amplifier of *n*-6 PUFA in human diets.

The socio-economic consequences of this expansion are profound. Large-scale agribusiness operations increasingly dominate soybean and oil-palm regions, driving land consolidation and reducing access for smallholders [39,40]. Rural economies become tightly coupled to volatile global commodity markets, heightening vulnerability to price shocks [41]. Mechanized soybean farming systems reduce rural employment, while oil-palm plantations often rely on precarious labour conditions [42]. These dynamics reshape local livelihoods, weaken traditional land-tenure systems, and intensify conflicts over land rights [43]. In regions such as Brazil, small farmers are frequently displaced as land is converted to large-scale soybean plantations, exacerbating unemployment and food insecurity in already vulnerable communities [44].

The socio-cultural burdens are equally significant. Oilseed expansion frequently overlaps with Indigenous territories and traditional farming landscapes, leading to displacement, erosion of customary land use, and loss of agrobiodiversity [45,46]. As monocultures replace diversified farming systems, traditional food cultures and ecological knowledge decline. The transformation of forests, savannahs, and mixed agroecosystems into industrial monocultures alters long-standing cultural relationships with land and undermines community resilience [47].

Ecologically, oilseed expansion is a major driver of deforestation, habitat loss, and greenhouse-gas emissions. Soybean cultivation contributes to land-use change in the Amazon, Cerrado, and Gran Chaco, while oil-palm expansion drives deforestation in Indonesia, Malaysia, and increasingly Latin America and West Africa [34,35,48]. These processes accelerate biodiversity decline, fragment ecosystems, and release large quantities of carbon from forests and peatlands [49]. In Brazil alone, soybean cultivation covered ~36 million hectares in 2019—an area roughly equivalent to the entire territory of Germany [29]. Intensive cultivation also contributes to soil degradation, nutrient depletion, and pesticide runoff, placing additional pressure on freshwater ecosystems [50]. Brazil has become one of the world’s largest consumers of herbicides, further contributing to species loss and environmental degradation [44].

**Table 2:** Global production volumes and cropland use for major oilseeds and vegetable oils.

Category	Production (Latest Global Estimates)	Cropland/Plantation Area	Notes
**Overall quantities**			
Total oilseeds [1,2,3]	~675–680 million tons	>300 million ha	Includes soybean, rapeseed, sunflower, peanut, cottonseed, palm kernel, copra
Total vegetable oils [1,6]	~234 million tons	—	Palm + soybean + rapeseed + sunflower ≈ 80% of global output
**Soy**			
Soybeans [1,2,3]	~420 million tons	~144 million ha	Dominant global oilseed
Soy for animal feed [29]	>300 million tons	~110–115 million ha	75–80% of global soy
Soy for biodiesel/renewable diesel [44,51]	Growing share of ~70 million tons of soybean oil	~10–12 million ha	Strongest growth in US, Brazil, EU
Soybean oil [1,6]	~70 million tons	Linked to soybean acreage	~20% of soybean economic value
**Other oils**			
Palm oil [1,6,8]	~80–81 million tons	~30 million ha	Largest single vegetable oil
Rapeseed oil [1,2,3]	~32–33 million tons	~44 million ha	Major crop in EU, Canada, China
Sunflower oil [1,2,3]	~22–23 million tons	~28 million ha	Concentrated in Ukraine, Russia, EU

Table Legend: Production data are derived primarily from the USDA Foreign Agricultural Service Oilseeds: World Markets and Trade reports and the USDA PSD Online database [23,31]. Land-use estimates are based on FAOSTAT cropland statistics and OECD-FAO Agricultural Outlook 2025–2034 projections [24,25,33]. Values are rounded to the nearest million metric tons or million hectares and represent the latest available global estimates for the 2024/25–2025/26 marketing years. Minor discrepancies between datasets reflect differences in reporting methodologies, harvested-area definitions, and marketing-year boundaries.

Together, these socio-economic, socio-cultural, and ecological burdens illustrate how global oilseed expansion has become a central driver of planetary change. Understanding this context is essential for evaluating the nutritional and biochemical implications of modern dietary patterns, particularly the dramatic rise in LA intake associated with industrial seed oils. In 2010 the FAO/WHO Expert Consultation already emphasized the importance of balancing *n*-6 and *n*-3 PUFA intake for metabolic health [14], a theme that becomes increasingly relevant in light of the agricultural transformations described above.

Within this broader context, the global rise of industrial seed oil has fundamentally altered the composition of modern diets. The dramatic increase in LA intake—driven by soybean, sunflower, safflower, and maize oils—has shifted the balance of dietary polyunsaturated fatty acids in ways that carry significant metabolic and physiological implications. These biochemical considerations form the basis for the following section.

Against this backdrop, the inclusion of these ecological and socio-economic considerations is not intended to shift the manuscript toward a broader political narrative, but rather to contextualize the biochemical and nutritional implications of modern PUFA exposure. The unprecedented rise in global seed-oil production is the primary upstream driver of contemporary LA intake, and understanding this structural transformation is essential for interpreting the metabolic and clinical patterns discussed in the following sections.

Last but not least, it is important to emphasize that the metabolic considerations discussed in this manuscript relate to dietary linoleic acid exposure and HUFA balance rather than to specific food categories. The term “seed oils” is used descriptively to denote major dietary sources of LA, not to imply inherent harmfulness. The health effects of *n*-6 PUFA–rich oils depend on the broader dietary pattern, the accompanying food matrix, background *n*-3 intake, and individual metabolic context. Accordingly, the discussion in this manuscript should not be interpreted as a categorical recommendation to avoid particular oil types, but rather as an exploration of how varying LA exposure influences HUFA balance within different nutritional environments.

### 2.3. Biochemical Rationale for a Nutritional Strategy Reducing n-6 and Increasing n-3 PUFA Intake

As alluded to earlier [6] there are good biochemical reasons to reduce dietary *n*-6 PUFA loads and increase *n*-3 PUFA supply in case of elevated *n*-6 HUFA levels measured in cell membranes. One of the first scientists to draw attention to this interrelationship was Bill Lands [6]. His central theses on PUFAs emphasize the critical balance between *n*-6 and *n*-3 fatty acids. Excessive intake of *n*-6 PUFAs displaces *n*-3 PUFAs from tissues, promoting inflammatory processes and potentially increasing the risk of chronic diseases, whereas restoring this balance through diet could help prevent a broad range of adverse health outcomes. The biochemical reasons are as follows:

#### 2.3.1. Biochemical Competition of PUFA Families

Between PUFA families there is biochemical competition in terms of their metabolism as well as competitive dynamics regarding their membrane incorporation. Shared metabolic pathways of *n*-6 and *n*-3 PUFAs give rise to competition for the same desaturase and elongase enzymes, influencing the production of long-chain HUFAs. In this context, an increase in the production of *n*-6 HUFAs has an impact on their membrane incorporation: Elevated *n*-6 HUFA production (especially arachidonic acid, C20:4*n*-6, AA) reflects a dominance of *n*-6-derived lipids in cell membranes, which predisposes to pro-inflammatory lipid-mediator signaling [6].

#### 2.3.2. Health Risks of Excessive *n*-6 PUFA Availability Can Be Counteracted by Targeted Intake of *n*-3 PUFAs

An increased intake of *n*-3 PUFAs and the resulting enrichment of cell membranes can counteract the aforementioned *n*-6 PUFA-related risks. The *n*-3 HUFAs eicosapentaenoic acid (C20:5*n*-3, EPA) and docosahexaenoic acid (C22:6*n*-3, DHA) promote an anti-inflammatory shift because they give rise to less potent eicosanoids and to lipid mediators (Specialized Pro-Resolving Mediators, SPMs) that actively resolve inflammation and support tissue repair (e.g., resolvins, protectins, and maresins). In essence, increased *n*-3 PUFA intake improves HUFA balance. Moreover, increasing *n*-3 PUFA intake while simultaneously reducing *n*-6 PUFA intake shifts the HUFA profile toward an even more anti-inflammatory and cardio-protective state [6].

As an additional practical measure, oils with a higher oleic acid content (C18:1*n*-9, OA)—such as high-oleic canola or sunflower oil—may help reduce dietary LA exposure when used in place of conventional seed oils (e.g., soybean, sunflower, safflower, or maize oil); however, their usefulness depends on a concurrent reduction in total *n*-6 PUFA intake and does not substitute for the limited endogenous conversion of ALA to EPA and DHA.

#### 2.3.3. Reducing *n*-6 PUFA Intake Is Essential if *n*-6 PUFA-Dominated Imbalance Prevails

Of note, while endogenous *n*-6 HUFA levels remain high, the effectiveness of increasing *n*-3 PUFA intake can only be ensured if dietary *n*-6 PUFA intake is reduced as extensively as possible—at least during the initial phase of the nutritional strategy proposed here. This is particularly relevant given the overwhelming dietary presence of LA in processed foods and seed oils, and consequently in the membranes of individuals consuming the so-called Western diet. As Lands emphasized, the therapeutic window for dietary PUFAs is essentially closed [6]. In addition, HUFA turnover is relatively slow, as reviewed elsewhere [6]. However, a sharp and sustained reduction in *n*-6 PUFA intake ultimately enables the displacement of *n*-6 HUFAs from membranes and allows for more effective incorporation of *n*-3 HUFAs.

#### 2.3.4. Proposed Therapeutic Principles Are Based on the Biochemical Rationale of Bill Lands

In short, Bill Lands’ PUFA theory posits that the tissue *n*-6/*n*-3 ratio drives metabolic and inflammatory processes and thereby influences chronic disease risk [1]. Consequently, dietary choices can be guided by explicit measures of this balance (*n*-6 HUFA scores) [6]. With this reasoning, he shifted the focus from total fat and saturated fatty acid intake toward PUFA composition and balance. In doing so, he highlights that lowering *n*-6 PUFA intake is as important as increasing *n*-3 PUFAs—an aspect often overlooked in mainstream nutrition advice.

The stratification of *n*-6 HUFA Scores into physiologically meaningful zones is grounded in Lands’ competitive HUFA-incorporation model [6] and validated through global HUFA distribution data. Lands demonstrated that *n*-3 and *n*-6 fatty acids compete for the same enzymatic pathways, resulting in predictable shifts in tissue HUFA composition as dietary *n*-6 intake increases [52,53]. This competition produces a continuum in which lower *n*-6 HUFA Scores reflect greater availability of *n*-3 HUFA substrates, whereas higher scores indicate progressive displacement of *n*-3 HUFA by *n*-6-derived species. The mathematical basis for translating dietary patterns into expected HUFA profiles was formalized by Lands and Lamoreaux, who showed that the relative difference between *n*-3 and *n*-6 intakes reliably predicts the resulting HUFA balance [54].

Population-level data corroborate these mechanistic predictions. In the global survey by Stark et al., regions with sustained marine food consumption—such as Japan, Iceland, and Mediterranean populations—cluster consistently within the 40–50% *n*-6 HUFA range, corresponding to a balanced HUFA pattern [55]. In contrast, Western dietary patterns, characterized by high linoleic acid intake and low long-chain *n*-3 availability, exhibit *n*-6 HUFA Scores exceeding 60%, with the United States, United Kingdom, and Germany frequently surpassing 70% [55,56]. These extreme values align with Lands’ earlier observations on the health implications of modern *n*-6-dominant diets [57] and with the recognized cardiometabolic risks associated with low omega-3 status [56]. Together, these mechanistic and epidemiological data justify the defined HUFA-zones since this framework integrates dietary exposure, biochemical competition, and global biomarker evidence into a coherent, biologically meaningful classification. Table 3 depicts reference ranges for *n*-6 HUFA Scores, their interpretation and their occurrence in representative populations.

The reference ranges for *n*-6 HUFA scores depicted in Table 3 are intended as a conceptual framework for understanding how dietary *n*-6 and *n*-3 intakes shape tissue HUFA composition, rather than as prescriptive clinical recommendations. Although observational and mechanistic evidence supports the plausibility of these relationships, no clinical trials have yet tested dietary interventions explicitly designed to achieve defined HUFA zones. The proposed ranges should therefore be interpreted as hypothesis-generating guidance rather than as validated therapeutic targets.

The therapeutic principles derived from Lands’ biochemical rationale are as follows: Policy and clinical guidance should aim for sufficient intakes of both PUFA families, while membrane balances should be assessed rather than administering high *n*-6 PUFA loads in isolation. It is essential that intake does not fall below minimum thresholds to avoid essential fatty acid deficiency (Table 4).

Classical essential fatty acid deficiency syndromes can be prevented with very low intakes of polyunsaturated fatty acids. Lands reported that approximately 0.2–0.3% of energy (≈0.5–0.7 g/day) of *n*-3 PUFAs and about 1% of energy (≈2–3 g/day) of *n*-6 PUFAs are sufficient to avoid neurological and dermatological manifestations of deficiency [3,58]. These minimal requirements are consistent with the deficiency thresholds originally characterized by Holman in the foundational EFAD studies [59]. In a later analysis, Lands distinguished these minimal physiological requirements from population-based intake standards and proposed EAR, RDA and UL values for linoleic acid of ~0.1%, ~0.5% and ~2% of energy, respectively [1]. However, Lands also emphasized that these physiological minimum intakes should not be used as practical dietary targets. Instead, he recommended maintaining intakes slightly above these thresholds (Table 4)—approximately 0.6–1.0 g/day (0.3–0.5% En) of total *n*-3 PUFAs, typically composed of ~0.4–0.6 g/day ALA plus ~0.2–0.4 g/day EPA + DHA, and 2–4 g/day (1–2% En) of LA—based on a reference energy intake of ~2000 kcal/day, to support a favorable tissue HUFA balance and an *n*-6/*n*-3 ratio of 1–4:1 [3,58].

It is important to note that the EAR and RDA values proposed by Lands in 2014 [1] do not represent revised minimal requirements. Instead, they reflect population-based statistical intake standards derived from distribution modeling and biochemical markers, whereas the classical minimal intakes reported in 2005 [3] and 2008 [58] refer to the prevention of overt clinical deficiency symptoms. The two concepts therefore describe different physiological endpoints and are not directly comparable. The practical intake range (functional adequacy) occupies a different conceptual space as well. It does represent a physiologically meaningful zone above both. These practical ranges reflect the intakes that reliably support optimal metabolic function, maintain appropriate tissue fatty acid composition, ensure sufficient availability of ALA and long-chain *n*-3 PUFAs (EPA + DHA), and preserve a balanced *n*-6/*n*-3 ratio. Numerically, these values exceed both the minimal deficiency-prevention thresholds and the EAR/RDA estimates because they are designed to capture functional sufficiency rather than statistical adequacy or clinical deficiency avoidance.

Of note, minimal requirements, practical intake ranges, and population-based intake standards originate from different scientific and institutional traditions. Minimal requirements are derived from biomedical and clinical research, whereas practical intake ranges represent expert-level functional estimates proposed by researchers such as Lands. In contrast, EAR, RDA and UL values are normally established by national or international nutrition authorities (e.g., NASEM/IOM within the Dietary Reference Intake framework, EFSA, or D-A-CH committees) through formal consensus processes.

Lands’ discussion of EAR/RDA/UL in his 2014 paper [1] should therefore not be interpreted as an attempt to replace or supersede the responsibilities of regulatory bodies. Rather, it represents a conceptual translation of his physiological data into the structure of the Dietary Reference Intake (DRI) system, illustrating how classical fatty acid physiology could be mapped onto population-based intake categories. His values thus serve as an expert-derived analytical framework, not as official dietary recommendations.

**Figure 1:**
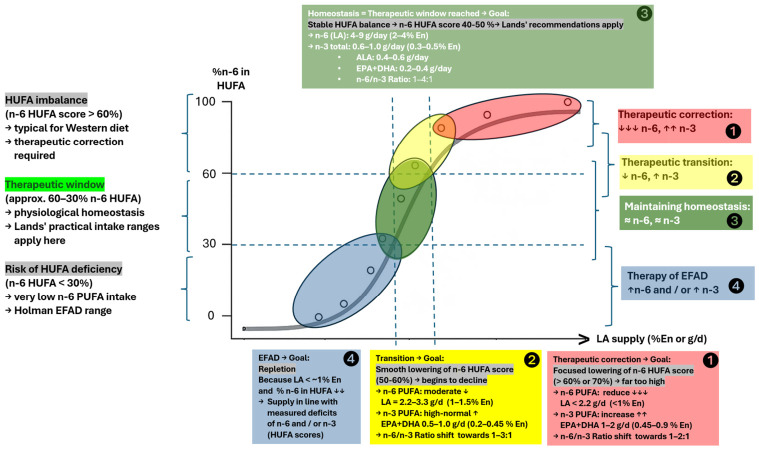
Conceptual model of HUFA status, physiological zones, and phase-specific PUFA intake strategies. Legend: Conceptual representation of the *n*-6 HUFA score across four physiological and therapeutic zones, arranged from right to left along the sigmoid curve: (1) the therapeutic correction zone, characterized by elevated *n*-6 HUFA levels and requiring targeted reduction in *n*-6 PUFA intake together with substantially increased *n*-3 PUFA supply; (2) the transition zone, in which moderate lowering of *n*-6 intake and high-normal *n*-3 intake facilitate progressive normalization of HUFA balance; (3) the homeostatic therapeutic window, where *n*-6 and *n*-3 inputs are balanced and Lands’ practical intake ranges apply (approximately 2–4 g/day LA and 0.6–1.0 g/day total *n*-3 PUFAs, including 0.4–0.6 g/day ALA and 0.2–0.4 g/day EPA + DHA); and (4) the EFAD zone, representing essential fatty acid deficiency and requiring repletion of *n*-6 and/or *n*-3 PUFAs according to measured deficits. The model illustrates that appropriate PUFA intake targets depend on an individual’s HUFA status, with more intensive modulation of *n*-6 and *n*-3 intake required during correction of an imbalance, and more moderate, stable intakes are suitable once homeostasis is restored. This figure represents a conceptual model derived from Lands’ HUFA-competition framework and has not been validated in clinical trials; it is intended for hypothesis-generating purposes only. Abbreviations: EFAD, essential fatty acid deficiency; HUFA, highly unsaturated fatty acids; PUFA, polyunsaturated fatty acids. In the context of PUFA intake, arrows indicate the relative direction and magnitude of change: ↑ = increase, ↑↑ = marked increase, ↓ = decrease, ↓↓ = marked decrease, ↓↓↓ = strong reduction. In the context of %*n*-6 in HUFA, arrows indicate the percentual presence of *n*-6 HUFAs in cell membranes: ↓↓ = marked decrease.

Although Lands typically models homeostatic HUFA balance at LA intakes of around 1–2% of energy (Table 4), several expert groups consider higher levels to remain physiologically safe. Current evidence suggests that adverse effects are unlikely below approximately 4% of energy from LA, which supports the broader intake range used in Figure 1. Analyses of recovered data from the Minnesota Coronary Experiment [60] and the Sydney Diet Heart Study [61] indicate that intakes of approximately 3–4% of energy from linoleic acid represent the highest exposure range consistently supported by neutral safety signals in randomized controlled trials, whereas substantially higher intakes (10–16% En) were associated with less favorable outcomes. Thus, ≈4% En reflects the upper range for which RCT-based safety evidence is available, rather than a prescriptive limit. Moreover, it is important to note that all these practical intake ranges represent guidance for individuals who are already within the physiological “therapeutic window” of HUFA homeostasis. During active therapeutic correction of an existing HUFA imbalance, however, temporarily lower *n*-6 PUFA intakes and substantially higher *n*-3 PUFA intakes may be appropriate to accelerate normalization of the HUFA profile before returning to the steady-state maintenance ranges described above and in Figure 1.

Last, it is important to emphasize that the intake thresholds and HUFA-based “therapeutic windows” discussed in this section are derived from Lands’ biochemical competition model and from global HUFA distribution data. They are not intended to replace or contradict existing dietary guidelines, which are based on different conceptual frameworks and do not incorporate HUFA profiling or competitive PUFA dynamics. The proposed thresholds should therefore be interpreted as hypothesis-generating estimates of the dietary conditions under which HUFA balance is expected to shift, rather than as evidence-based clinical cut-offs. Further research is required to determine how these conceptual ranges relate to guideline-based recommendations in real-world settings.

The dietary principle can be summarized as follows: ensure adequate intake of both PUFA families while continuously assessing and adjusting HUFA balance. Measurement of the endogenous HUFA profile is particularly informative, as it reveals the degree of imbalance and provides a rational basis for modifying the nutritional regimen. In practical terms, *n*-6 PUFA intake should remain within an appropriate range—neither excessive nor so low as to risk deficiency—while *n*-3 PUFA intake is increased to support rebalancing of the HUFA ratio. This approach could be particularly relevant if elevated levels of *n*-6 PUFAs are detected in individuals who follow a Western diet.

#### 2.3.5. Measuring Blood *n*-6 and *n*-3 HUFAs—And Their Ratio—Is an Indispensable Health Risk Assessment (HRA) Measure

The non-energetic effects of PUFAs, their relation to non-communicable diseases, the extraordinarily high intake of *n*-6 PUFAs (as LA) within the “Western diet”, the mutual metabolic interference of *n*-6 and *n*-3 PUFAs and thus the large differences in the individual PUFA availability should be considered acceptable reasons for their routine blood evaluation today. The relevance of these interferences found in both experimental and clinical trials calls for a standardized analytical procedure, which is already established in several laboratories worldwide, and for a discussion on possible reimbursement within national medical systems [62]. The findings resulting from these measurements should be included in routine health risk assessments. A health risk assessment (HRA) typically consists of a questionnaire, an assessment of health status, and personalized feedback on actions that can be taken to reduce risk, maintain health, and prevent disease. It is a valuable tool for promoting health awareness and informed decision-making. Laboratory values can also be included in an HRA. These values add important information about a person’s health status. Parameters already in use include cholesterol levels, blood glucose levels, HbA1c, liver and kidney function tests, inflammatory markers (e.g., C-reactive protein), or vitamin and mineral levels. There is a rising body of evidence now that the easily measured proportion of PUFAs in blood cells (which in general represents/monitors average dietary intake) can be a valid risk indicator as well and may prevent diseases or save even lives if adequate measures are implemented [63]. To an extent this is already done by assessing the endogenous *n*-6/*n*-3 PUFA-ratio [64] or by means of the omega-3 index (EPA + DHA in red blood cells) [65], but these measures do not provide sufficient information on *n*-6 PUFAs. As an alternative, therefore, the percentage of *n*-6 HUFAs in the total amount of HUFAs of whole blood (*n*-3/*n*-6 HUFA balance score) could be considered [3,58]. This score is used to quantify the impact of dietary fats on tissue HUFA composition and appears to be most suitable if used as an integral and valuable part of a HRA, applicable in preventive as well as acute medicine. A higher percentage of *n*-6 HUFA is associated with a more pro-inflammatory lipid mediator profile, while a lower percentage—ideally below 50%—suggests a more balanced, anti-inflammatory profile.

In this context, the proposal to incorporate HUFA profiling into routine health assessment is therefore intended as a hypothesis-generating concept rather than as a prescriptive clinical recommendation. While several studies underscore the prognostic value of accurately measured fatty acid profiles—including the omega-3 index, the omega-6/omega-3 ratio, and HUFA-based predictors of cardiometabolic risk [62,63,64,65]—no clinical trials or health-system evaluations have yet demonstrated improved outcomes through routine HUFA measurement. Further research is required to determine clinical utility, cost-effectiveness, and the feasibility of implementing standardized HUFA profiling in real-world settings.

#### 2.3.6. An Excess of *n*-6 HUFAs in Cell Membranes Could Pose a Health Risk—But Remains Difficult to Interpret Without Concurrent *n*-3 PUFA/HUFA Assessment

The potential health implications of elevated *n*-6 HUFA levels—particularly arachidonic acid (AA)—are supported by strong mechanistic plausibility. However, these mechanisms should not be interpreted as evidence of established clinical causality. The following section therefore distinguishes mechanistic hypotheses from observational associations and findings from randomized controlled trials (RCTs).

Mechanistically, an excess of *n*-6 HUFAs can reduce *n*-3 HUFA incorporation, narrow the therapeutic window for *n*-6 PUFA intake when *n*-3 availability is low, alter membrane fluidity, and shift intracellular signaling and gene expression toward more pro-inflammatory pathways. AA serves as the precursor for prostaglandins and leukotrienes that promote inflammation, immune suppression, thrombosis, fibrosis, and vasoconstriction [66]. These biochemical effects have led several authors, including Lands [6], to propose that elevated *n*-6 HUFA levels may contribute to cardiovascular disease, metabolic syndrome, and other chronic inflammatory conditions.

Observational evidence, however, is heterogeneous. High dietary *n*-6 PUFA loads have been linked to a wide range of pathological conditions, including cancer, cardiovascular disease, Alzheimer’s disease, asthma, COPD, MASLD, gallstone disease, obesity, type 2 diabetes, osteoporosis, depression, diverticulitis, and premature aging [67,68,69,70]. The “Western diet,” with its disproportionately high LA content, may therefore act as a catalyst that initiates or perpetuates underlying pathologies, including autoimmune diseases [1].

At the same time, several large prospective cohort studies and meta-analyses report that higher dietary or circulating linoleic acid (LA) levels are associated with lower risks of total mortality, cardiovascular mortality, cancer mortality, and type 2 diabetes [51,71,72,73,74,75,76,77,78]. A recent systematic review and meta-analysis of 38 cohorts (811,069 participants) found that high LA intake was associated with reduced all-cause, cardiovascular, and cancer mortality (RR 0.87–0.89) [77]. Mendelian randomization analyses similarly suggest that genetically predicted higher LA levels are associated with lower diabetes risk and improved lipid profiles, although no benefit for ischemic heart disease was observed [79].

However, when we look at RCTs, the evidence we find there remains limited. No trial has directly linked membrane *n*-6 HUFA levels to clinical endpoints. Two older dietary intervention trials reported increased cardiovascular mortality when LA intake was raised without increasing *n*-3 PUFA intake [60,61]. The Minnesota Coronary Experiment [60] and the Sydney Diet Heart Study [61] are historically important but methodologically complex trials, and their findings have been the subject of substantial debate. Their inclusion in this manuscript is therefore intended to illustrate the potential consequences of increasing LA intake in the absence of adequate *n*-3 PUFA availability, rather than to serve as definitive evidence of harm. More recent meta-analyses generally report neutral or protective associations of *n*-6 PUFA intake with cardiovascular outcomes, reducing LDL-cholesterol and improving cardiometabolic risk markers [71,78,80]—particularly when replacing saturated fat. However, these analyses aggregate trials that typically did not assess HUFA status, concurrent *n*-3 PUFA intake, or the competitive metabolic interactions central to Lands’ framework. As such, both the older and more recent bodies of evidence must be interpreted within the limitations of their respective methodological contexts. Conversely, multiple trials have demonstrated that when *n*-3 PUFA levels are adequate—the balance of both PUFA families optimizes lipoprotein metabolism, inflammatory responses, insulin sensitivity, and neurological outcomes [81,82,83,84].

Other clinical trials of *n*-3 PUFA supplementation have produced inconsistent results for diseases such as cancer and cardiovascular disease [85,86,87], further illustrating the complexity of interpreting PUFA effects without considering baseline HUFA status.

Taken together, the mechanistic rationale for considering HUFA balance remains strong, but clinical evidence is heterogeneous. These divergent findings likely reflect differences in baseline *n*-3 status, dietary context, and the absence of HUFA profiling in most cohorts. As Lands emphasized [6], biological effects of individual PUFAs cannot be interpreted in isolation but must be understood within the competitive interplay between the *n*-6 and *n*-3 families. Without quantifying *n*-3 HUFAs (e.g., RBC or phospholipid EPA and DHA), reported associations between *n*-6 PUFA intake, plasma levels, or triacylglycerol fractions and health outcomes risk misattribution—and the reverse holds true for *n*-3 PUFA associations. The near-universal absence of *n*-6 PUFA intake assessment and HUFA profiling in clinical trials may therefore obscure true biological effects [62].

#### 2.3.7. Pathogenetic Significance of Lipodystrophy, Ectopic Visceral Fat and Insulin-Resistance in Light of an *n*-6/*n*-3 PUFA and HUFA Imbalance

Against the backdrop of seemingly contradictory evidence, it may appear difficult to engage intellectually with an extension of Lands’ theories. It is therefore important to emphasize that Lands’ framework represents one of several conceptual models used to interpret PUFA metabolism and cardiometabolic risk. Alternative perspectives—some of which were outlined above—highlight, for example, the generally neutral or protective associations of total *n*-6 PUFA intake reported in large cohort studies and meta-analyses, oxylipin-centered interpretations of inflammatory signaling, or guideline-based assessments prioritizing overall PUFA replacement of saturated fats. The present manuscript thus employs Lands’ model as a conceptual lens rather than as a universally accepted explanatory system. Notably, emerging insights into the pathophysiology of metabolic syndrome and related organo-metabolic disease processes are opening new perspectives that may help reconcile these differing interpretations [88,89].

The development of diabetic comorbidities—more broadly situated within the spectrum of non-communicable diseases—can be conceptualized as the emergent consequence of several mutually reinforcing pathophysiological mechanisms that together disrupt systemic metabolic homeostasis. A reduced capacity of adipose tissue to expand and safely store surplus energy results in a state of relative lipodystrophy, in which subcutaneous adipocytes fail to buffer postprandial lipid fluxes and increasingly release free fatty acids and lipid intermediates into the circulation [90,91,92,93]. This chronic oversupply of fatty acids imposes a metabolic burden on peripheral tissues and constitutes a major driver of insulin and leptin resistance by impairing receptor signaling [88,94,95,96], altering membrane lipid composition [94], and activating serine/threonine kinases that inhibit insulin receptor substrate function [88,94,95,96,97,98]. Hormonal resistance, in turn, accelerates unrestrained lipolysis and promotes the redirection of lipids toward non-adipose organs [88,90,94,96,99,100]. The resulting ectopic lipid deposition in liver, skeletal muscle, myocardium, pancreas, and vascular tissue marks a critical transition point at which metabolic overload is converted into structural and functional cellular injury [88,90,94,96,99,100]. Within these tissues, the combination of elevated FFA flux, accumulation of toxic lipid species such as ceramides and diacylglycerols, mitochondrial overload with incomplete β oxidation, and sustained activation of stress pathways (Table 5)—including unfolded protein response, JNK signaling, inflammasome activation, and impaired autophagic flux—creates a lipotoxic environment that destabilizes organellar integrity and triggers apoptotic and necro-inflammatory cascades.

Visceral ectopic fat including metabolic dysfunction–associated steatotic liver disease (MASLD)—rather than overall obesity—is a central driver of cardiometabolic disease [111,112] and therefore represents a therapeutic target. It is well established that visceral fat accumulation is associated with insulin resistance, dyslipidemia, systemic inflammation, and vascular dysfunction, which collectively define the metabolic syndrome. Importantly, both diabetic and nondiabetic individuals presenting with metabolic syndrome exhibit increased all cause and cardiovascular mortality [113,114] and we are beginning to understand the role that “lipodystrophy” and “lipotoxicity” play in this process in more detail.

Moreover, emerging data indicate that this metabolic environment also reshapes the gut microbiome. Individuals with lipodystrophy exhibit reduced microbial diversity and compositional shifts that resemble the dysbiosis observed in metabolically unhealthy obesity, despite the absence of excess body fat. This suggests that “adipose tissue dysfunction,” whether due to deficiency (lipodystrophy) or excess (obesity), may converge on a common gut microbial signature, as recently reviewed by Colangeli et al. [115]. The mechanisms likely involve altered bile acid metabolism, changes in intestinal lipid flux, chronic low-grade inflammation, and endocrine disturbances that affect gut barrier integrity and microbial ecology, consistent with the broader pathophysiological framework outlined by Fourman and Grinspoon [116].

Once established, dysbiosis can further aggravate insulin resistance. Microbial products such as lipopolysaccharide (LPS) activate innate immune pathways (e.g., TLR4), amplifying systemic inflammation and impairing insulin signaling in liver, muscle, and adipose tissue [117,118]. Altered production of short-chain fatty acids, secondary bile acids, and branched-chain amino acid metabolites can disrupt enteroendocrine function, GLP-1 secretion, hepatic lipid handling, and peripheral glucose uptake [119,120,121]. Thus, the microbiome becomes not merely a passive marker but an active contributor to worsening metabolic dysfunction.

This creates the possibility of a self-reinforcing cycle: lipodystrophy → ectopic fat → microbiome disruption → insulin resistance → further ectopic fat accumulation and adipose tissue stress, with each component of this cycle supported by mechanistic and clinical evidence as reviewed elsewhere [115,116]. Although the full loop has not yet been empirically demonstrated in humans as a single causal chain, each individual link is supported by mechanistic and clinical evidence.

A high dietary *n*-6/*n*-3 polyunsaturated fatty acid (PUFA) ratio may act as an upstream amplifier of this cycle, primarily by shifting the balance of bioactive lipid mediators toward arachidonic-acid-derived inflammatory pathways and by increasing the metabolic demand for *n*-3 long-chain PUFAs [122,123]. Excess *n*-6 PUFA intake favors the formation of pro-inflammatory eicosanoids, whereas insufficient *n*-3 PUFA reduces the availability of anti-inflammatory and pro-resolving lipid mediators. This imbalance can exacerbate adipose tissue inflammation and impair hepatic lipid metabolism.

In parallel, dietary fat quality strongly influences gut barrier integrity and microbial ecology. High-fat, saturated-fat-rich dietary patterns are consistently associated with dysbiosis, increased intestinal permeability, and metabolic endotoxemia [124,125,126,127]. Omega-3 PUFAs, in contrast, have been shown to modulate microbial composition and reduce endotoxin-driven inflammation [122,128]. Within this broader framework, it is biologically plausible that an unfavorable *n*-6/*n*-3 ratio may further modulate these processes by limiting the availability of *n*-3-derived anti-inflammatory and pro-resolving mediators, even though direct evidence linking PUFA imbalance to gut microbial disruption remains limited.

Taken together, these observations support a unifying hypothesis: adipose tissue dysfunction, ectopic lipid deposition, gut microbial alterations, and systemic insulin resistance may form a mutually reinforcing network of disturbances, potentially modulated by dietary fatty acid composition. This framework provides a coherent basis for future mechanistic studies and may help explain why metabolic derangements in lipodystrophy are so severe and difficult to reverse.

**Figure 2:**
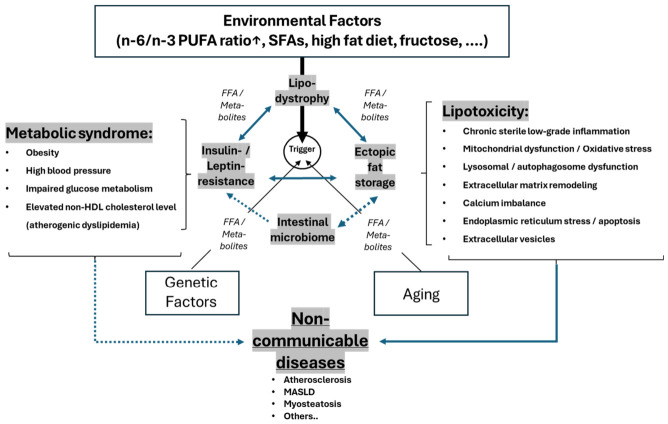
Integrated pathophysiological model linking lipodystrphy, insulin–leptin resistance, ectopic fat storage and intestinal dysbiosis to the development of non-communicable diseases. Legend: Environmental factors, aging, and genetic predisposition interact with a quadrilateral architecture of metabolic failure comprising (i) impaired adipose tissue expandability with relative lipodystrophy, (ii) insulin–leptin resistance, (iii) ectopic fat storage with lipotoxic stress, and (iv) intestinal dysbiosis. These processes increase circulating free fatty acids and metabolites, drive hormonal resistance, and promote lipid redirection into non-adipose organs, where mitochondrial overload, incomplete β-oxidation, ER stress, impaired autophagy, and inflammasome activation culminate in lipotoxic injury. Dysbiosis further contributes through barrier dysfunction, altered bile-acid signaling, metabolic endotoxemia, and shifts in microbial metabolites that amplify systemic inflammation. Together, these disturbances give rise to organ-specific complications such as MASLD, myosteatosis, atherosclerosis, cardiomyopathy, β-cell dysfunction, nephropathy, neuropathy and others. Abbreviations: PUFA = polyunsaturated fatty acid; FFA = free fatty acid; MASLD = metabolic dysfunction–associated steatotic liver disease; β-oxidation = beta-oxidation; ER = endoplasmic reticulum. Solid arrows indicate well-established or bidirectional interactions supported by consistent evidence; dashed arrows represent hypothesized or less-confirmed relationships.

As depicted in Figure 2, the development of diabetic comorbidities is conceptualized as the emergent consequence of the mutually reinforcing pathophysiological mechanisms addressed already. When considered in their full interdependence, these disturbances delineate a coherent quadrilateral architecture of metabolic failure, in which nearly each component amplifies the dysfunction of the others and thereby accelerates systemic metabolic deterioration. More downstream, metabolic syndrome and lipotoxicity are further important pathophysiological drivers that develop from this interdependence and can also influence and reinforce each other. However, within this conceptual framework, lipotoxicity may be regarded as an upstream driver of metabolic dysfunction, whereas metabolic syndrome increasingly appears as a downstream epiphenomenon that reflects, rather than initiates, the underlying pathophysiological injury.

Impaired adipose tissue expandability reduces the capacity for safe triglyceride sequestration and increases the spillover of free fatty acids and lipid intermediates into the circulation, thereby overwhelming peripheral tissues with substrates that exceed their oxidative and storage capacities. This chronic lipid influx activates a spectrum of intracellular stress pathways depicted in Table 5.

These effects converge with the core mechanisms of lipodystrophy, insulin–leptin resistance, and ectopic lipid deposition, lowering metabolic flexibility (ability to oxidize the optimal substrate at the right time) and accelerating the transition from nutrient excess to organellar stress caused by lipotoxicity and sterile inflammation. This framework underscores that the progression of diabetic complications could even be driven less by hyperglycemia per se than by a systemic failure of lipid buffering, intracellular stress adaptation, and inter-organ communication—with lipotoxic pathways arising from spillover of free fatty acids and lipid intermediates standing in no way inferior to glucose-driven mechanisms.

Environmental drivers—including an elevated *n*-6/*n*-3 PUFA ratio, saturated fatty acids, a high fat diet, and fructose—potentiate the turn-over within the “quadrilateral architecture” by enhancing adipose tissue inflammation, increasing hormonal resistance (insulin, leptin) as well as intestinal dysbiosis and suppressing mitochondrial adaptability among other mechanisms, while genetic predisposition and aging exacerbate declines in mitochondrial function, proteostasis, and immune resolution (Figure 2). From this integrated network of disrupted lipid partitioning, maladaptive signaling, and organellar stress responses arise the metabolic syndrome and the canonical organ complications of lipotoxicity, including MASLD/MASH, myosteatosis, atherosclerosis, diabetic cardiomyopathy, β-cell dysfunction, nephropathy, and neuropathy.

In recent years, Bill Lands’ theses have been extended into this field. A comparatively high endogenous availability of *n*-6 PUFAs (particularly ARA) relative to *n*-3 PUFAs may promote ectopic fat development. Increased inflammatory mediator production and alterations in lipid metabolic signaling pathways appear to favor fat storage in liver, muscle, and visceral tissue [9]. Again, these effects depend strongly on the endogenous *n*-6/*n*-3 PUFA ratio and thus on the metabolic context. A high ratio promotes pro-inflammatory signaling (e.g., via leukotrienes and prostaglandins), which is associated with insulin resistance and fat redistribution [129,130].

Several human and experimental studies directly examine the impact of the *n*-6/*n*-3 PUFA ratio on metabolic outcomes, ectopic fat, and cardiometabolic risk—going beyond traditional PUFA vs. SFA comparisons [131]. In mice, high fat diets enriched with *n*-6 PUFAs promote weight gain, insulin resistance, and hepatic steatosis, whereas *n*-3 PUFAs reduce fat mass and improve hepatic pathology [132]. Conversely, lowering the dietary *n*-6/*n*-3 ratio from 30:1 to 5:1 modulates inflammation related gene expression and improves metabolic markers in obese mice [133]. In Mexican adults, dietary *n*-6/*n*-3 ratios ≥ 10:1 were associated with higher hepatic steatosis index (HSI) values and increased odds of fatty liver [134]. Human studies further show that lowering the *n*-6/*n*-3 ratio improves blood lipids, reduces tissue lipid accumulation [81], and enhances insulin clearance in obese youth—independent of weight loss or insulin secretion [135]. A meta-analysis reported that low *n*-6/*n*-3 ratios significantly reduce triglycerides and increase HDL C, with benefits strengthening over time [136].

Collectively, these findings indicate that the *n*-6/*n*-3 HUFA balance may be an important determinant of lipid metabolism and ectopic fat accumulation, extending Lands’ central thesis. This is of public-health relevance, as Western dietary patterns often exhibit ratios of 10–20:1—substantially higher than the frequently cited evolutionary range of approximately 1–4:1. Several mechanistic pathways may explain these associations:

- Inflammatory mediators: Arachidonic acid (ARA)–derived eicosanoids modulate macrophage and adipocyte inflammatory activity and contribute to dysregulated lipid handling in metabolic tissues [137,138]. ARA-derived prostaglandins and leukotrienes activate NF-κB and JNK signaling, thereby amplifying inflammatory tone and impairing insulin-receptor signaling [139,140].
- Insulin resistance: A chronic, inflammation-prone, *n*-6-dominant milieu disrupts insulin action and increases free-fatty-acid flux into ectopic depots [141,142]. Elevated ARA availability is consistent with enhanced TLR4-dependent inflammatory signaling in adipose tissue [143,144]. This pathway converges on IKKβ/JNK activation, promoting inhibitory IRS-1 serine phosphorylation and impaired insulin action [141].
- Lipid species and ApoB: High plasma levels of free ARA and ARA-rich triglyceride species correlate with dyslipidemia and ApoB overexpression, aligning with lipidomic signatures associated with hepatic and vascular fat accumulation [145]. Long-chain PUFA-enriched phospholipids—including ARA-containing species—can activate SREBP1c and suppress PPARα, thereby increasing VLDL secretion and reducing fatty-acid oxidation [146].
- Signal transduction: As reviewed recently [6], *n*-6 PUFAs modulate multiple intracellular pathways. ARA-derived oxylipins engage pro-inflammatory MAPK cascades, while low EPA/DHA availability limits the formation of pro-resolving mediators (resolvins, protectins, maresins), prolonging inflammatory signaling [140,147].
- Transcriptional regulation: Low *n*-3 HUFA availability reduces PPARα activation and thereby limits hepatic fatty-acid oxidation [146]. High *n*-6 HUFA availability diminishes suppression of SREBP1c and NF-κB, enhancing lipogenesis and inflammatory signaling [139]. Conversely, EPA/DHA supplementation activates hepatic AMPK and improves metabolic stress responses; thus, low EPA/DHA availability is expected to reduce AMPK activation, favoring lipogenic and stress-related pathways [148,149].
- Membrane lipid partitioning: Excess *n*-6 PUFAs alter membrane phospholipid composition and microdomain organization, influencing receptor-dependent inflammatory signaling [94,144,150]. Reduced EPA/DHA incorporation disrupts lipid-raft dynamics and weakens anti-inflammatory receptor pathways such as GPR120 [151]. Long-chain PUFA-containing phospholipids and their oxidation products orchestrate raft-associated signaling events that bias cells toward pro-inflammatory activation [152], contributing to downstream disturbances in metabolic regulation, including impaired insulin sensitivity.
- Atherosclerotic lesions and endothelial dysfunction: A reduced *n*-6/*n*-3 ratio decreases atherosclerotic lesions in apoE(−/−) mice, likely via anti-inflammatory effects of *n*-3 HUFAs [153]. Dietary manipulation studies show that high ratios (e.g., 20:1) increase endothelial oxidative stress, elevate E-selectin and von Willebrand factor, and impair vascular function, whereas lower ratios (1:1–5:1) improve endothelial reactivity and inflammatory status [154]. These findings suggest that excess *n*-6 relative to *n*-3 PUFAs shifts the endothelium toward a pro-inflammatory, pro-atherogenic phenotype.
- Mitochondrial dysfunction and energetic imbalance: Mitochondrial dysfunction is a recognized contributor to endothelial impairment [155]. High *n*-6/*n*-3 ratios are associated with increased inflammation, oxidative stress, and endothelial dysfunction [154]. However, direct causal studies linking impaired *n*-6 HUFA balance, mitochondrial dysfunction, and endothelial dysfunction remain limited, and current evidence is primarily mechanistic and indirect.
- Fibrogenic pathways: Progressive liver fibrosis in MASLD/MASH results from chronic hepatocellular injury, oxidative stress, inflammatory activation, and sustained stellate-cell stimulation, culminating in TGF-β–mediated collagen deposition and architectural remodeling [156]. Elevated *n*-6/*n*-3 ratios and reduced *n*-3 HUFA availability are associated with exacerbation of these processes by enhancing hepatic oxidative stress and pro-inflammatory signaling, thereby promoting stellate-cell activation and potentially accelerating the transition from steatosis to fibrosis [157].

Although the evidence is not fully consistent and some findings remain controversial [158], the overall body of data supports the hypothesis advanced here. High dietary *n*-6/*n*-3 PUFA ratios are associated with unfavorable lipid profiles and increased visceral adiposity in humans, while animal models consistently show that *n*-6-dominant diets promote hepatic fat accumulation unless counterbalanced by *n*-3 PUFAs. Conversely, *n*-3 HUFAs exert protective effects by enhancing fatty-acid oxidation, improving lipid handling, and attenuating inflammatory signaling.

In summary, high endogenous *n*-6 PUFA availability can promote ectopic fat deposition when *n*-3 PUFA availability is relatively low. The relationship is likely nonlinear and influenced by diet, genetics, and endogenous PUFA metabolism. Practically, optimizing the dietary and endogenous *n*-6/*n*-3 PUFA balance—ideally toward a ratio of approximately 1–4:1—may help prevent ectopic fat accumulation and its metabolic consequences. This can be achieved by reducing dietary *n*-6 PUFA intake (e.g., sunflower, maize, safflower oils) and increasing *n*-3 PUFA intake (e.g., fish oil, flaxseed oil).

However, an important controversy must be acknowledged: when *n*-6 PUFA intake or membrane levels are evaluated in isolation, some studies report an inverse association with ectopic fat (i.e., higher *n*-6 correlates with less ectopic fat) [158]. In contrast, when the *n*-6/*n*-3 PUFA ratio is assessed, the opposite pattern emerges. This discrepancy has been discussed in detail elsewhere [6] and definitely requires further clarification.

Despite some contradictory findings, a coherent hypothesis can nevertheless be advanced by synthesizing fundamental biochemical insights with the integrated pathophysiological model shown in Figure 2. The conceptual framework proposed by Lands provides a biochemically grounded lens through which the metabolic syndrome appears not merely as a cluster of cardiometabolic risk factors, but as the systemic manifestation of a long-standing imbalance in PUFA metabolism. A chronically elevated dietary *n*-6/*n*-3 ratio shifts the eicosanoid milieu toward proinflammatory, vasoconstrictive, and oxidation-prone signaling pathways, thereby lowering the threshold for chronic inflammatory activation across multiple organ systems [3,58,159]. Within this perspective, the metabolic syndrome can be understood as a downstream indicator syndrome that more likely reflects the systemic impact of upstream lipotoxicity, while still potentially exerting its own modulatory effects on the emergence of organo-metabolic disorders unified by impaired inflammatory resolution, endothelial dysfunction, and disrupted cellular energetics.

Lipotoxicity represents the central mechanistic link in this cascade. The accumulation of fatty acids and their oxidized derivatives in non-adipose tissues promotes mitochondrial dysfunction, reactive oxygen species generation, endoplasmic reticulum stress, and activation of innate immune pathways—processes that are remarkably conserved across cardiac, hepatic, renal, skeletal muscle, and neural tissues [88,160,161,162]. Baidya et al. recently framed heart failure with preserved ejection fraction (HFpEF) as a systemic cardiometabolic syndrome driven by insulin resistance, visceral adiposity, endothelial dysfunction, and chronic low-grade inflammation [163]. Although their review does not explicitly address PUFA biology, the mechanistic constellation they describe aligns closely with the downstream consequences of a long-term *n*-6-dominant dietary pattern: proinflammatory eicosanoid signaling, impaired production of specialized pro-resolving mediators, oxidative stress, and reduced mitochondrial efficiency.

Comparable mechanistic signatures are evident in other organo metabolic syndromes (Table 6). MASLD is characterized by PUFA dependent lipid peroxidation and the accumulation of oxidized linoleic acid metabolites (OXLAMs), which activate inflammatory and fibrogenic pathways [164,165]. Chronic kidney disease (CKD) progression is strongly influenced by endothelial dysfunction, microvascular rarefaction, and inflammatory lipid mediators, all of which are modulated by cellular lipid composition [166]. Neurodegenerative disorders such as Alzheimer’s disease exhibit altered membrane lipid architecture, impaired resolution of inflammation, and increased lipid peroxidation—features that map directly onto Lands’ framework of PUFA driven inflammatory imbalance [10,167,168,169]. Even the polycystic ovary syndrome (PCOS) demonstrates PUFA-linked amplification of insulin resistance, androgen excess, and systemic inflammation [170,171].

The integrated framework presented in Figure 2 aligns even closely with emerging translational insights from oncology, as highlighted in the recent JAMA review on obesity and cancer [179]. Both perspectives converge on the notion that adipose tissue dysfunction, ectopic lipid deposition, hormonal resistance, and microbiome-derived inflammatory cues constitute a shared pathophysiological architecture that extends beyond classical metabolic disease. The JAMA review underscores how lipotoxic stress, chronic low-grade inflammation, altered adipokine signaling, and dysbiosis-driven metabolites create a systemic milieu that promotes not only cardiometabolic complications but also tumor initiation and progression. This parallel reinforces the broader relevance of the quadrilateral model of metabolic failure, suggesting that the same disturbances—impaired lipid buffering, mitochondrial overload, ER stress, and barrier dysfunction—form a common soil from which both non-communicable metabolic diseases and obesity-associated cancers emerge.

Taken together, these converging lines of evidence support a unifying interpretation: the metabolic syndrome may represent the systemic expression of a PUFA modulated inflammatory and energetic imbalance, while organo-specific syndromes such as HFpEF, MASLD/MASH, CKD, neurodegeneration, or PCOS reflect tissue specific vulnerabilities to this shared biochemical disturbance. Lipotoxicity functions as the mechanistic core, whereas the clinical phenotype is determined by the organ system most susceptible to oxidative and inflammatory stress. In this sense, Lands’ concept of a “diet driven inflammatory imbalance” provides a plausible and integrative root cause for a broad spectrum of modern chronic diseases.

## 3. What Are the Inferences Derived for the Development of New Nutritional Strategies?

The term malnutrition is usually applied to describe a diet that does not meet physiological requirements in terms of quantity and/or quality. In general, this refers to deficits in nutrition (i.e., undernutrition). However, an unbalanced high intake of energy sources, minerals (e.g., iron or sodium) or vitamins has also become subsumed under this umbrella term. This type of malnutrition is also called overnutrition (hyperalimentation), a form of malnutrition in which the intake of nutrients is excessive: the amount of nutrients exceeds the amount required for metabolism, normal growth, development, and normal function of organs or systems. Thus, not only does a deficiency of specially identified nutrients play a pathogenetic role in the development of diseases, but also, an excessive availability of dietary substrates has a disease-inducing and perpetuating impact. The curiosity with the topic in question is that an oversupply of *n*-6 PUFAs was either not acknowledged or neglected as a possible pathogenetic factor up until today.

To give this form of malnutrition the level of attention it deserves, it might be helpful to refer to it by a special term—for example, “dysnutrition”, a term used here descriptively to denote a specific qualitative imbalance rather than a classical under- or overnutrition state. This form of malnutrition therefore describes less an over- or undersupply of energy and nutrients, which can be easily identified by assessing the content of the diet or the supplements used. Rather, it refers to a hidden imbalance of individual substrates, such as the imbalance between *n*-6 and *n*-3 PUFAs in the diet. A related disease-adapted nutritional strategy must therefore not only focus on the correction of any identified deficits or excesses but must also ensure the normalization (here the reduction) of significantly increased substrate availabilities concealed in food.

In accordance with the hypotheses of Lands, we would pose the following requirements for the therapy of *n*-6 PUFA related “dysnutrition”:

- Besides an adequate supply of energy, protein, vitamins, and minerals, it is important to reduce the over-availability of pathogenetically acting dietary substrates (next to the *n*-6 PUFAs examples of these might also include certain saturated fats, simple sugars and sodium). This may require new nutritional concepts. Even the best-designed nutritional therapy to compensate for identified substrate deficits (e.g., of *n*-3 PUFAs) will not achieve its optimal effectiveness if over-availabilities of other nutrients are not corrected and if dietary habits—which favor an excessive intake of *n*-6 PUFAs—are not fundamentally and sustainably changed. This may require the temporary reduction in a substrate to “near zero”, even if this substrate is acknowledged to be essential.
- This approach’s legitimacy results from the dietary over-availability of *n*-6 PUFAs in the form of LA, which has arisen in the context of industrialized food production, whereby seeds and seed oils became an industrial food staple.
- However, before starting therapy for *n*-6 PUFA related “dysnutrition”, it is mandatory that *n*-6 PUFA over-availability is laboratory proven (by the *n*-6 HUFA score) and that there is at least one co-morbidity associated with it (e.g., atherosclerosis, obesity, type II diabetes, or others).
- Once *n*-6 PUFA supply is restricted, adequate monitoring tools should be implemented as a safety and compliance measure to avoid essential fatty acid deficiency (EFAD) and to assess follow-up compliance if chronic diseases are to be approached and non-compliance is expected. Such an approach then also appears to be necessary in the long term as sufficient maintenance of appropriate PUFA and HUFA intakes must be continuously ensured to either prevent malnutrition (deficits) or the return of “dysnutrition” (*n*-6 PUFA over availability).
- Repeated measurements of PUFAs could also be applied as follow-up monitoring after successful normalization of the *n*-6 PUFA availability, working as a surrogate indicator of clinical improvement throughout the course of the disease.
- The easily measured proportion of *n*-6 HUFAs (and *n*-3 HUFAs) in the total amount of HUFAs in blood or erythrocytes can be a valuable risk indicator or monitoring tool and may prevent diseases or even save lives if adequate measures are implemented. So, the percentage of *n*-6 HUFAs in the total amount of HUFAs of whole blood could be considered a valuable health risk assessment (HRA) measure for preventive medicine.
- In the case of “dysnutrition”, dietary counseling would have to be expanded to include advice on the avoidance of *n*-6 PUFA-containing foods (e.g., use low LA oils like olive oil, high oleic sunflower oil and so on), provided an increased *n*-6/*n*-3 PUFA ratio can be demonstrated in individual patients and provided that this finding can be related to a defined morbidity.
- The assessment of blood HUFAs by laboratory measurements should be established as an initial risk and prognostic risk factor of *n*-6 PUFA triggered diseases as part of any nutritional assessment.

## 4. “Corner Stones” for a Nutritional Concept to Normalize a Pathological *n*-6/*n*-3 PUFA Ratio

### 4.1. Prerequisites and Objectives for the Successful Application of the Nutritional Concept

As with all nutritional interventions, the first step would be to screen and assess for “dysnutrition” using standard procedures. However, this should be completed by a targeted anamnesis for co-morbidities that could be associated with a dysregulation of PUFA metabolism (e.g., cardiovascular diseases, obesity, type II diabetes, cancer and others). If there is evidence of relevant co-morbidity—and thus evidence of the first special requirement—a laboratory chemical analysis of the entire PUFA profile in erythrocytes should be conducted. In this test, a second special prerequisite is required, namely a significantly increased “*n*-6 PUFA load”, usually recognizable by the increased ARA concentration seen as higher % HUFAs as *n*-6. A third requirement is testing for a partial or even complete *n*-3 PUFA deficiency—in particular concerning ALA (C18:3*n*-3), EPA and DHA—which is usually accompanied by a consecutive reduction in the omega-3 index and % HUFA as *n*-3 and an increase in the *n*-6/*n*-3 ratio. Especially here it is important to realize that a reduced endogenous *n*-3 HUFA availability in combination with even normal *n*-6 HUFA levels also leads to an increased *n*-6/*n*-3 PUFA ratio that may trigger pathological consequences as well.

If the laboratory findings are as described above, they suggest that an existing *n*-6 PUFA oversupply and/or an *n*-3 PUFA deficiency lead to a “through-metabolism” of the ingested LA to ARA. This preferential metabolism of *n*-6 PUFAs appears to be promoted in particular by the lack of competitive inhibition of the desaturases used jointly by *n*-6 and *n*-3 PUFAs. The lack of *n*-3 PUFAs allows the unrestricted utilization of the pathway by the *n*-6 PUFAs and enables the accumulation of ARA and consequently the lipid mediators derived from it.

The aim of the nutritional concept described here is to increase the omega-3 index to a value > 8 and to decrease the % HUFAs as *n*-6 to less than 50% while the individually measured levels for *n*-6 PUFAs must not fall below the threshold for essential fatty acid deficiency.

### 4.2. Recommended Therapeutic Nutritional Approach

The therapeutic suggestions outlined here do not correspond explicitly to vegetarian, vegan, Mediterranean, or other established dietary patterns. Nevertheless, several of the proposed measures may overlap partially or implicitly with such diets. In addition, some mitigation strategies have been described that help identify and reduce dietary sources contributing to excessive linoleic acid (LA) intake while increasing the supply of *n*-3 PUFAs [32,180]. Although Lands extensively characterized the biochemical and kinetic principles governing HUFA balance, to our knowledge he did not publish a formalized dietary protocol in the scientific literature. Practical dietary guidance associated with his work appears primarily on his personal website rather than in peer-reviewed publications. Accordingly, the dietary concept presented here aims primarily at the normalization of a demonstrably pathological fatty acid status—specifically, the correction of deranged endogenous *n*-6 and *n*-3 PUFA levels. It is important to emphasize that not only nutrient deficiencies (e.g., insufficient *n*-3 PUFA intake) but also nutrient excess (e.g., excessive *n*-6 PUFA intake) can have pathological consequences. Furthermore, potentially harmful fatty acids such as industrial or heat-generated trans fatty acids—formed during inappropriate processing or cooking methods such as frying or grilling—should also be avoided. In detail, the following measures comprise the proposed therapeutic approach:

#### 4.2.1. Significant Increase in Oral *n*-3 PUFA Intake

Increasing *n*-3 PUFA intake via diet or supplementation can help with a variety of pathologies, e.g., type 2 diabetes, obesity, metabolic syndrome and CVD [85,181,182]. This is relevant with regard to health maintenance and disease prevention across the entire lifespan. Therefore, more attention should be paid to their adequate intake. This can be approached in various ways:

- Aiming for a targeted increase in the oral intake of seawater fish (e.g., salmon, mackerel, herring, sardines). Wild sources are preferred, since farmed sources may be lower in *n*-3 PUFAs than wild sources and may contain higher *n*-6 PUFA (LA) levels. Freshwater fish are a less favorable option as they eat and store a small amount of *n*-3 PUFAs only.
- Aiming for a targeted increase in the oral intake of algae and seaweed products. There is an increasing selection available.
- Aiming for a targeted increase in *n*-3 PUFAs via the intake of supplements. This can be achieved through fish oil–based or algal oil–based products. Upon closer examination, however, it becomes evident that uniform recommendations in this area are difficult to establish.

Following the theories of Bill Lands [6] and their application by Hibbeln J.R. et al. [123], it becomes clear that the global variability in dietary intakes of *n*-6 and *n*-3 fatty acids is substantial. This variability not only shapes tissue HUFA compositions and associated disease risks but also determines the required dosage of *n*-3 HUFA intake. Based on these considerations, a target value of 40–50% *n*-3 HUFAs in tissue was defined, as this level reflects typical dietary intakes in Japan—intakes that appear to protect approximately 98% of the population from disease risks. Under these conditions, a healthy dietary allowance for *n*-3 HUFAs would be estimated at roughly 3.5 g/day for a 2000 kcal U.S. diet and about 2.2 g/day for German diets. Notably, this allowance could likely be reduced to one-tenth of these amounts if dietary *n*-6 fatty acid intake were substantially lowered [123]. Such a differentiated approach, however, has not yet been incorporated into current dosage recommendations.

Other statements on dosage recommendations suggest that daily intakes of around 1 g EPA + DHA are required to achieve physiologically meaningful effects, with higher doses producing more pronounced responses. Evidence from clinical guidelines and intervention studies supports the use of approximately 1 g/day EPA + DHA when specific physiological targets are intended. The American Heart Association recommends a daily intake of about 1 g EPA + DHA for individuals with established coronary heart disease, preferably from fish, while supplements are considered an appropriate alternative [183]. Similarly, the GISSI-Prevenzione trial demonstrated that supplementation with 1 g/day EPA + DHA significantly reduced all-cause mortality and the risk of sudden cardiac death following myocardial infarction [184]. Moreover, Calder’s synthesis of mechanistic and clinical evidence indicates that physiologically relevant increases in plasma and tissue *n*-3 PUFA levels, as well as measurable anti-inflammatory effects, generally require intakes of around 1 g/day or higher [122].

However, many commercially available supplements and several national recommendations provide daily amounts well below 1 g EPA + DHA, and these intakes remain markedly lower than those typically achieved in populations with regular fish consumption [185]. A recent review of quantitative intake recommendations [186] reported a median recommended intake of 313 mg/day EPA + DHA for adults, with 250 mg/day being the most frequently cited value. The authors emphasize that even these modest recommendations are difficult to achieve through the habitual dietary patterns of Western populations and may therefore necessitate supplementation.

The partly inconsistent recommendations for *n*-3 PUFA supplementation, together with the established mechanistic understanding of PUFA metabolism [6], highlight the need to formulate several guiding principles that may serve as a basis for further discussion.

It may be advisable to precede any initiation of *n*-3 PUFA supplementation with an assessment of membrane-bound fatty acid profiles in blood. As described by Hibbeln et al. [123], the variability in *n*-6 PUFA burden across populations—and likely also between individuals—is substantial. This variability is accompanied by correspondingly different *n*-3 PUFA requirements, which should be matched to the pre-existing *n*-6 PUFA load. For this reason alone, empirically fixed or “calculated” *n*-3 PUFA supplementation strategies are no longer appropriate.

Moreover, establishing the baseline fatty acid status is essential for determining whether supplementation is indicated at all, whether support for maintenance therapy is sufficient, or whether a more intensive intervention with substantially higher doses is required.

In addition, evaluation of existing *n*-6 PUFA and HUFA levels is necessary to decide whether dietary reduction in this fatty acid family should be recommended.

Only once these questions have been clarified through a simple laboratory analysis does it become meaningful to consider an appropriate strategy and to define the *n*-3 PUFA dose for supplementation. It follows naturally that the resulting dosage recommendations may differ considerably, and that a “one-size-fits-all” approach is unlikely to be useful. It should also always be considered that PUFA requirements can, as far as possible, be met through natural dietary sources.

If supplements are used, however, it is important to ensure that they are of high quality, not oxidized (i.e., low peroxide and anisidine values), and as free as possible from contaminants such as heavy metals (e.g., mercury). Algal oil does not need to originate from marine environments; it can be produced in well-controlled cultures and is therefore typically low in pollutants. It is also suitable for vegetarians and vegans.

#### 4.2.2. Significant Reduction in Oral *n*-6 PUFA Intake

According to the rationale provided by Lands and others (see above), the optimal target availability of LA in foods—defined as the amount required to prevent essential fatty acid deficiency—is generally set at 1–2% of daily energy intake, corresponding to approximately 2.5–5 g/day (based on optimal energy intake relative to body weight). An “*n*-6 PUFA–reducing approach” (clearly below this target availability) must be maintained over a considerable period of time—certainly longer than a few months. Of note, the half-life of LA is approximately 680 days, or roughly two years [187], as reviewed elsewhere [32]. This implies that replacing about 95% of the LA stored in the body with “healthier” fatty acids may take up to six years. In view of these findings, it becomes evident that chronic LA over-loading from seed oils can be detrimental to overall health, making sustained reduction in LA intake essential.

Thus, for the first period of the diet proposed here, a truly radical “lifestyle change” must be implemented, aimed at markedly reducing the most identifiable *n*-6 PUFA sources in the diet. This is anything but easy to achieve—but it is probably the only way to bring about relevant relief from an existing *n*-6 PUFA burden. After laboratory confirmation of the normalization of the fatty acid profile, the “stringency” of the nutritional concept could be eased, which would allow the intake of *n*-6 PUFAs up to an upper limit of 2%E. However, a certain “lifestyle change” will have to be maintained throughout the rest of life in order not to lose the healthy fatty acid status that has been achieved. Various *n*-6 PUFA burdens in “normal” eating habits must be considered and avoided, as the *n*-6 PUFAs are ubiquitously hidden in the so-called “Western diet”. Some recommendations to avoid any “open” or “hidden” *n*-6 PUFA intakes are summarized below:

- The intake of *n*-6 PUFA-rich seed oils should be avoided. They are not only “visibly” integrated in cooking but also “hidden” through inclusion in nearly all processed foods from industrial manufacturers. Thus, the lowest LA-containing source of fats would be the preferred fats of choice for lowering the LA burden in the diet—e.g., olive oil, high-oleic sunflower oil and some rapeseed oil [32]. These “admissible oils” still may present with an unfavorable LA to ALA ratio but the absolute proportions and amounts of *n*-6 PUFAs are comparatively low. Nevertheless, caution is also advised with olive oil since a nearly 10-fold wide variability in the percentage of LA (ranging from 3% to 27%) was demonstrated [188]. In addition, commercial olive oil can be adulterated with seed oils high in *n*-6 PUFAs [189]. The use of coconut oil is, however, possible without restriction as part of the diet described here. Coconut oil is very low in LA although it does not have the essential fat-soluble vitamins that animal fats have [32]. If animal sources of fat are used, these should come exclusively from grass-fed animals. In addition to containing the lowest LA content, the latter sources of fats also provide fat-soluble vitamins A, D and K2 [32].
- In order to avoid hidden *n*-6 PUFA uptake, industrially produced foods should be avoided since it cannot be certified that they are free of *n*-6 PUFAs.
- Milk and meats from ruminants can be consumed in moderation if desired [32]. Ruminants include cows/beef, buffalo, sheep/lambs, goats, deer, elk and many other wild animals. They are reported to have low LA levels in their meat and milk, regardless of what they eat [190]. This is in contrast to animals with a single stomach, such as chickens and pigs, which experience an increase in LA concentrations in their tissues when fed an LA-rich diet, including maize and soybean, similar to the process observed in humans [191]. Thus, intake of chicken or porcine (pig) meat in particular, should be limited [32].
- Ideally, the diet should relate to raw materials, products and recipes listed on the Lands website “Essential Fatty Acids Home Page” [192], which present those with the highest possible *n*-3 PUFA and the lowest possible *n*-6 PUFA contents.

#### 4.2.3. Strict Adherence to All Other Known Recommendations for a “Healthy Diet”

The diet should generally aim for fat reduction, moderate calorie reduction and increased protein intake. In addition, attention should be paid to an adequate intake of fiber, phytochemicals, vitamins, trace elements and essential as well as conditionally essential nutrients.

Fat reduction helps to minimize hidden *n*-6 PUFAs by reducing the caloric load and specific sources of LA. The creation of energy-dense foods promotes hyperalimentation. A classic example is “fast food”, which allows us to quickly eat high-calorie and fatty food before a feeling of satiety arises. The nexus between “fast food” and an oversupply of *n*-6 PUFAs therefore represents an ominous combination. A moderate caloric reduction helps to burn deposited *n*-6 PUFAs after they have been hydrolyzed and released from fat depots. The re-esterification of these lipolytically released *n*-6 PUFAs should be avoided if possible, although it cannot be prevented completely. To prevent from consuming “trans fats,” fried and grilled foods should be avoided as much as possible.

Protein intake should be kept high. At the expense of fat and also to a certain extent of carbohydrates, protein supply may be at the higher end of recommendations, which are moving towards higher intakes than previously suggested. A protein intake of up to 1 g/kg body weight can be aimed for, provided that kidney function is normal. Inflammation-related cachexia (due to underlying illness or therapeutic procedures) should also be counteracted by an increased protein supply. Such a protein intake can be achieved using low-fat products such as low-fat curd cheese, yoghurt and cottage cheese (all with a fat content of 0.2%). Skimmed milk is available as a source of fluid and protein—here too, the fat content should not exceed 1.5%. Not least, another advantage of enhanced protein intake is an appetite-suppressing effect.

A targeted reduction in hidden and open sugars (especially simple sugars like fructose) is desirable.

A high proportion of fruit and vegetables in the diet is particularly important for an adequate supply of fiber, phytochemicals, vitamins, trace elements and essential as well as condition-essential nutrients.

### 4.3. Implementation of an Exercise Program

Such a program should be an integral part of a “concept,” as exercise triggers positive immunological and metabolic processes that promote overall health. In addition, an increase in energy consumption helps to support weight loss and thus a reduction in “deep” fat deposits by promoting oxidative use of lipolytically released fats and by opposing their re-esterification and deposition. However, the exercise program is a supportive measure only that underpins the dietary intervention to correct the existing “dysnutrition”, as it cannot intervene causally. The cause of the pathomechanisms in question is probably the increased *n*-6 HUFA balance, which leads to metabolic and immunological changes that cannot be addressed causally by exercise therapy.

## 5. Strengths and Limitations

This work offers several distinct strengths. It provides an integrated synthesis of emerging organo-metabolic pathophysiology, an area that is receiving increasing attention in the context of non-communicable disease development. By assembling current evidence on the principal drivers of these processes and presenting them within a coherent conceptual framework, the review aims to clarify relationships that are often discussed in isolation. These pathophysiological considerations are further connected to well-established findings from basic research on the biological actions of PUFAs and HUFAs, allowing preliminary exploration of how these mechanistic insights may intersect with organo-metabolic dysfunction. In addition, contemporary data on the production and processing of seed-oil crops are consolidated and examined in relation to potential pathophysiological mechanisms, as well as their socio-economic, cultural, and ecological implications. By framing these interrelations as an unresolved and insufficiently examined problem, the review seeks to sharpen awareness and encourage a reevaluation of current risk-assessment practices, which remain only marginally addressed in the public domain. From this synthesis, a potential nutritional strategy is proposed that, to our knowledge, has not previously been articulated with comparable focus or integrative breadth.

Several limitations must also be acknowledged. Although the mechanistic insights derived from basic research on PUFAs and HUFAs are widely regarded as established, most of the broader relationships discussed here remain associative rather than demonstrably correlative or causal. The inferences drawn from the assembled evidence therefore require further experimental and clinical validation before firm conclusions can be drawn. This need for empirical substantiation also applies to the proposed nutritional strategy, which should be regarded as a hypothesis-generating framework rather than a prescriptive recommendation.

## 6. Concluding Remarks

So what are we dealing with? We are dealing with a steady increase in the large number of non-communicable diseases (NCDs) and their impact on morbidity and mortality. At the same time, we are seeing a steady increase in the dietary availability of *n*-6 PUFAs in the “Western Diet,” which has its origins in the industrialized production and use of seed oils. A possible connection between these two developments has been established by findings from basic research, which we owe to scientists such as Bill Lands. Unfortunately, we lack proof that these are correlations rather than associations. We lack further and more in-depth insights into the very complex biochemical and immunological relationships, and we lack well-designed clinical studies that could corroborate the basic knowledge more reliably. Amid all this uncertainty, however, it remains plausible that this development may carry risks on a global scale that warrant closer scientific scrutiny. These considerations should be enough to prompt a discussion about how we want to continue dealing with this risk.

Bill Lands’ theses on the PUFA balance have been partly validated and partly challenged by opposing epidemiological evidence. When *n*-6 PUFAs are evaluated alone, studies often show that higher *n*-6 PUFA levels are linked to lower disease risk. But when the *n*-6/*n*-3 PUFA ratio is examined, this endpoint consistently emerges as a critical modifier, showing concordant associations between an increasing ratio and increasing disease risk. The key reason is that the *n*-6/*n*-3 PUFA ratio reflects the balance of opposing biochemical effects—whereas *n*-6 PUFAs alone may appear beneficial in isolation but obscure the antagonistic role they play when *n*-3 PUFAs are insufficiently available. Although the available evidence does not yet allow definitive causal hierarchies to be established, the current body of data consistently indicates that hyperglycemia and dysregulated lipid metabolism act through partially overlapping, mutually reinforcing pathways. This warrants a cautious interpretation of mechanistic claims while acknowledging the robustness of the overall pattern. The discourse on PUFA balance is of fundamental dietary relevance; however, its full exploration lies beyond the scope of this contribution and requires further in-depth evaluation.

Against this background, an elevated endogenous *n*-6/*n*-3 PUFA ratio can be understood as one potential driver within a multifactorial system affecting lipid metabolism and contributing to the pathogenesis of NCDs. Beyond the imbalance of PUFA or HUFA ratios, additional nutritional stressors—such as excessive availability of saturated fatty acids, high-fat and hypercaloric dietary patterns, and specific carbohydrates like fructose—further amplify metabolic strain. These exogenous dietary factors operate alongside genetic predispositions, epigenetic influences, and age-related processes as relevant determinants of disease risk. When viewed in an even broader context, the pathogenic consequences of impaired carbohydrate metabolism, which become apparent and effective through chronic hyperglycemia—including glycation (AGE-RAGE signaling), oxidative stress, PKC activation, inflammation, and endothelial dysfunction—represent only one dimension of metabolic damage. Accumulating evidence suggests that lipotoxicity constitutes an equally potent and still underappreciated driver of diabetic and metabolic comorbidities, operating through ectopic lipid deposition, mitochondrial overload, ceramide-mediated stress signaling, and inflammation. Recognizing the dual and convergent toxicity of glucose and lipids is therefore essential for contextualizing the contribution of PUFA imbalance within the wider metabolic network.

Building on the PUFA-related biochemical specifics, the socio-economic and socio-cultural dimensions, and the ecological burdens—addressed here and discussed in line with Bill Lands’ theses elsewhere [6]—a nutritional strategy can be proposed as follows:

- Avoid high-LA oils and hidden *n*-6 PUFA rich food sources: Eliminate or drastically reduce sunflower, maize, soybean, and grapeseed oils and consistently reduce the consumption of industrially processed foods or dietary products from industrialized animal farming.
- Increase EPA + DHA intake: Prioritize fatty fish (e.g., sardines, mackerel, salmon) or use purified fish oil (or algal oil) supplements.
- Monitor baseline HUFA status and changes in progress: Use HUFA balance, omega-3 index and red blood cell fatty acid profiles to track biochemical response over 8–12 weeks at least, or even for a longer period of time.

Finally it can be concluded as follows: A diet that radically reduces *n*-6 PUFA uptake and strategically increases *n*-3 PUFA supply might be a suitable intervention to rebalance the body’s deranged HUFA pool. This could lead to a metabolic shift from increased fat deposition (especially of ectopic fat) to increased fat oxidation and an immunologic shift from inflammation and immune suppression to anti-inflammation and immune-augmentation. Such a nutritional strategy could provide a positive metabolic effect through the direct action of *n*-3 PUFAs and HUFAs on signal transduction, gene expression, and cell membrane function. This could potentially mitigate the clinical sequalae of lipodystrophy, insulin-resistance and the development of ectopic fat. In turn, this could ultimately have a positive clinical impact by reducing the incidence of metabolic syndrome and the pathogenesis of lipotoxicity. It could also reduce the substrate for pro-inflammatory mediators and enhance the production of inflammation-resolving lipid mediators. Overall, this diet may help to lower chronic disease risk and boost metabolic health. But more experimental and clinical studies are required to further evaluate the nutrition strategy proposed here before broader recommendations for implementation can be made.

## Figures and Tables

**Table 1:** Approximate percentage of energy from linoleic acid (%En LA) across selected populations.

Population/Diet Pattern	Approx. %En LA	Basis of Estimate	Evidence Type
United States (NHANES adults)	~5–7%En ^a^	LA intake ~10–20 g/day; mean energy ~2000–2500 kcal/day	Approximated
Western Europe	~3–6%En ^b^	Regional LA intake ranges summarized by Adam [16]	Approximated
Traditional Mediterranean	~3–5%En ^c^	High MUFA (olive oil), moderate seed oil	Inferred
Japan (traditional/transitional)	~3–4%En ^d^	Lower seed oil use; higher marine fats	Approximated/inferred
Kitava (Melanesian horticulturalists)	~0.5–2%En ^e^	Oils/cereals contribute ~0.03% of energy; coconut dominant	Inferred
Other traditional groups (Hadza, Shuar, Tsimane, etc.)	~1–3%En ^f^	Minimal seed oils; wild/animal fats	Inferred

Table legend: Values are derived from reported LA intake, typical energy intake, or inferred from dietary structure. They should be interpreted as order-of-magnitude estimates rather than precise measurements. ^a^ United States: NHANES analyses indicate LA intakes of ~10–20 g/day, corresponding to ~4–7%En LA given typical energy intake. Consistent with FAO/WHO and AHA recommendations and high seed-oil consumption [14,15,17]. ^b^ Western Europe: LA intake generally lower and more variable than in the US, depending on sunflower/soybean oil use [16]. ^c^ Mediterranean diet: High MUFA (olive oil) and modest seed-oil use yield moderate LA intake [16]. ^d^ Japan: Lower seed-oil consumption and higher marine fat intake produce lower *n*-6 PUFA exposure [16]. ^e^ Kitava: Oils, cereals, and sugar contribute only ~0.03% of energy; coconut (low LA) is the main fat source [18,19]. ^f^ Other traditional groups: Dietary structure and biomarker profiles indicate low LA exposure; precise %En not reported [20,21].

**Table 3:** Reference Ranges for *n*-6 HUFA Scores Based on Lands’ Model and International HUFA Profiles.

*n*-6 HUFA Score	Interpretation	Representative Populations	Key References
**<40%**	Very low; EFAD-proximal; high *n*-3 HUFA availability	Traditional coastal populations	[3,7,55,57]
**40–50%**	Balanced HUFA pattern (“homeostasis zone”)	Japan, Iceland, Mediterranean	[7,54,55,58]
**50–60%**	Transition zone (*n*-6 dominant but modifiable)	Mixed dietary patterns	[7,52,58]
**>60%**	Markedly *n*-6 dominant	Western dietary patterns	[7,52,53,55,56]
**>70%**	Pathologically high *n*-6 dominance	USA, UK, Germany	[7,55,56,57]

Legend: The table summarizes characteristic ranges of the *n*-6 HUFA score as described in the HUFA-balance model developed by Lands and colleagues, together with representative population data from international HUFA profiling studies. The *n*-6 HUFA score reflects the measured proportion of *n*-6 highly unsaturated fatty acids (HUFA) within total HUFA in blood lipids and serves as an indicator of the competitive balance between dietary *n*-6 and *n*-3 fatty acids. Lower *n*-6 HUFA Scores (<40%) reflect traditional dietary patterns with high *n*-3 HUFA availability and represent the lower end of the physiological range, approaching the EFAD-proximal zone where *n*-6 scarcity becomes biochemically evident. Values around 40–50% are typically observed in populations with balanced long-chain PUFA status (e.g., Japan, Iceland, Mediterranean regions) reflecting populations with sustained marine intake. Scores above 60% indicate marked *n*-6 dominance typical of Western dietary patterns, and values exceeding 70% represent extreme *n*-6 predominance associated with low omega-3 status in industrialized nations. Reference ranges are supported by Lands’ mechanistic HUFA competition framework [52,53,54] and by global HUFA distribution data [55,56].

**Table 4:** Minimal physiological requirements, practical intake ranges, and population-based intake standards for essential fatty acids.

Parameter	*n*-3 PUFAs	*n*-6 PUFAs (Linoleic Acid)	Sources
Minimal intake to prevent classical deficiency symptoms	~0.2–0.3% En (≈0.5–0.7 g/day)	~1% En (≈2–3 g/day)	Lands 2005 [3]; Lands 2008 [58]
Practical intake range (functional adequacy)	~0.3–0.6% En (~0.6–1.0 g/day)	~1–2% En (~2–4 g/day)	Lands 2005 [3]; Lands 2008 [58]
Practical sub-ranges (*n*-3 PUFA composition)	ALA: ~0.4–0.6 g/day (0.2–0.3% En) EPA + DHA: ~0.2–0.4 g/day (0.1–0.2% En) *n*-6/*n*-3 ratio: 1–4:1	–	Lands 2005 [3]; Lands 2008 [58]
Estimated Average Requirement (EAR)	–	~0.1% En	Lands 2014 [1]
Recommended Dietary Allowance (RDA)	–	~0.5% En	Lands 2014 [1]
Tolerable Upper Intake Level (UL)	–	~2% En	Lands 2014 [1]

Legend: Minimal physiological requirements refer to the lowest intakes of essential fatty acids that prevent classical deficiency syndromes, including neurological manifestations for *n*-3 PUFAs and dermatological manifestations for *n*-6 PUFAs. Practical intake ranges represent the functional intake zones described by Lands, indicating levels that reliably support physiological sufficiency beyond the mere avoidance of deficiency. These include sub-ranges for ALA and EPA + DHA as well as the *n*-6/*n*-3 ratio. EAR (Estimated Average Requirement) denotes the average daily intake estimated to meet the requirement of 50% of healthy individuals in a population. RDA (Recommended Dietary Allowance) represents the intake sufficient to meet the needs of 97–98% of healthy individuals. UL (Tolerable Upper Intake Level) indicates the highest average daily intake that is unlikely to pose risks of adverse health effects. Percent of energy is based on a reference intake of ~2000 kcal/day. The table uses rounded values.

**Table 5:** Mechanisms Driven by Impaired Adipose Tissue Expandability and FFA/Lipid Metabolite Overload.

Category/Mechanism	Mechanistic Description	References
***Lipodystrophy/Adipose Expandability Failure***		
Loss of adipose storage capacity	Limited expandability → FFA spillover → ectopic fat and metabolic stress	[90,91,92,93]
Impaired adipocyte differentiation	Inflammation and lipotoxicity impair PPARγ → reduced adipogenesis	[90,92,93,94]
Adipocyte death and macrophage recruitment	Lipid-overloaded adipocytes → necrosis → macrophage infiltration	[90,92,93,101]
Hypoxia in hypertrophic adipose tissue	Hypertrophy → hypoxia → HIF-1α → fibrosis and inflammation	[90,94]
Adipokine dysregulation	Reduced adiponectin, leptin resistance → impaired metabolic regulation	[92,93,102,103,104]
***Insulin and Leptin Resistance***		
DAG-mediated PKC activation	DAG → PKCθ/ε → IRS serine phosphorylation → impaired insulin signaling	[88,94,95,96]
Ceramide-mediated Akt inhibition	Ceramides inhibit Akt/PKB → reduced glucose uptake	[88,94,95,96]
Impaired GLUT4 translocation	Lipid intermediates disrupt GLUT4 trafficking	[88,94,95,96]
AMPK suppression	High lipid availability reduces AMPK activity → impaired FA oxidation	[94,95]
Hypothalamic inflammation	FFA-induced ER stress and TLR4/JNK → central leptin/insulin resistance	[104]
Sympathetic dysregulation	Leptin resistance alters sympathetic tone → ↓ energy expenditure	[102,104]
***Metabolic Syndrome Pathways***		
Chronic low-grade inflammation	FFAs activate NF-κB → cytokines → systemic IR	[90,97,98,101]
TLR4-dependent inflammatory signaling	Saturated FFAs activate TLR4-dependent pathways	[97,98,101,105]
NLRP3 inflammasome activation	Ceramides, ROS, lysosomal stress → NLRP3 → IL-1β	[101,106]
Endotoxemia/gut permeability	Lipid-induced gut permeability → LPS-TLR4 activation	[105]
Fibrosis	Hypoxia and inflammation activate fibroblasts → ECM deposition	[90,92,93,107]
Hepatokines and myokines	FGF21, fetuin-A, myostatin → systemic IR	[100,105]
Systemic metabolic consequences	Combined pathways → MetS, MASLD, T2D	[100,105]
***Ectopic Fat Storage***		
Ectopic lipid accumulation	FFAs stored as DAGs, ceramides, acyl-CoAs → organ dysfunction	[88,90,94,96,99,100]
Altered membrane lipid composition	Sphingolipids/cholesterol alter membrane microdomains	[94]
Extracellular vesicles	Lipid-laden EVs propagate inflammatory signals	[108]
***Lipotoxicity***		
Acylcarnitine accumulation	β-oxidation overload → acylcarnitines → mitochondrial stress	[95]
Incomplete β-oxidation	High FFA flux → partially oxidized intermediates	[95,109]
Mitochondrial stress and ROS	Excess FFAs → ROS → oxidative damage	[90,109]
ER stress/UPR activation	Lipid overload → PERK/IRE1/ATF6 activation	[94,97,98,110]
Lysosomal dysfunction	Lipid accumulation impairs autophagy	[106]
JNK activation	Lipid intermediates activate JNK → IRS-1 inhibition	[94,97,98]
Randle cycle/substrate competition	Elevated FFA oxidation suppresses glucose oxidation	[88,94,95,96]
***Intestinal Dysbiosis***		
Microbial dysbiosis	High-fat states alter microbiota → ↑ LPS, ↓ SCFAs → systemic inflammation	[105]
Microbial metabolites	Dysbiosis-derived metabolites impair hepatic/adipose signaling	[105]
Gut barrier dysfunction	Lipid-induced inflammation weakens tight junctions → endotoxemia	[105]

Abbreviations: Akt/PKB = protein kinase B; AMPK = AMP-activated protein kinase; ATF6 = activating transcription factor 6; DAG = diacylglycerol; ER = endoplasmic reticulum; EVs = extracellular vesicles; FFA = free fatty acids; FGF21 = fibroblast growth factor 21; GLUT4 = insulin-responsive glucose transporter type 4; HIF-1α = hypoxia-inducible factor 1-alpha; IL-1β = interleukin-1 beta; IRE1 = inositol-requiring enzyme 1; IRS = insulin receptor substrate; JNK = c-Jun *N*-terminal kinase; LPS = lipopolysaccharide; MetS = metabolic syndrome; MASLD = Metabolic dysfunction–Associated Steatotic Liver Disease; NF-κB = nuclear factor kappa-light-chain-enhancer of activated B cells; NLRP3 = NOD-like receptor family pyrin domain containing 3; PERK = PKR-like ER kinase; PKC = protein kinase C; PPARγ = peroxisome proliferator-activated receptor gamma; T2D = type 2 diabetes; TLR4 = Toll-like receptor 4; UPR = unfolded protein response. Arrows indicate the relative direction and magnitude of change: ↑ = increase, ↓ = decrease.

**Table 6:** Mechanistic Overview of Organo-Metabolic Syndromes: Lipotoxicity and the Role of PUFA Imbalance.

Organometabolic Syndrome	Core Mechanisms	Lipotoxicity and the Role of PUFA Imbalance	Key Evidence
Heart Failure (HF)	Insulin resistance, endothelial dysfunction, microvascular inflammation, mitochondrial inefficiency	*n*-6-PUFA–derived pro-inflammatory eicosanoids may promote endothelial activation and systemic metaflammation; insufficient formation of specialized pro-resolving mediators (SPMs) may impair inflammatory resolution. Lipotoxic overload compromises myocardial energetics and contributes to HFpEF-like phenotypes.	Baidya 2026 [163]; Unger 2010 [160]; Ertunc 2016 [162]
Metabolic dysfunction–Associated Steatotic Liver Disease (MASLD)/Metabolic dysfunction–Associated Steatohepatitis (MASH)	Hepatic steatosis, oxidative stress, ER stress, inflammation, fibrosis	Oxidized linoleic acid metabolites (OXLAMs) accumulate in MASLD/MASH and may amplify hepatocellular stress and inflammatory signaling; free fatty acids promote lipotoxic pathways that drive inflammation and fibrogenesis.	Puri 2007 [164]; Feldstein 2004 [165]
Chronic Kidney Disease (CKD)	Endothelial dysfunction, microvascular rarefaction, oxidative stress, chronic inflammation	*n*-3 PUFAs show antifibrotic and lipotoxicity-attenuating effects in experimental models. In observational cohorts, lower *n*-3 PUFA status and reduced *n*-3:AA ratios correlate with adverse cardiometabolic profiles and have been linked to higher cardiovascular and renal risk. Higher *n*-3 intake is associated with slower progression of diabetic kidney disease.	Han 2023 [172]; Liboriussen 2025 [173]; Wan 2026 [174]; Koh 2024 [175]; Shima 2013 [176]
Neurodegenerative Disorders (NDD)	Neuronal oxidative stress, impaired resolution of inflammation, mitochondrial dysfunction, synaptic failure	Altered membrane PUFA composition increases susceptibility to lipid peroxidation; reduced SPM formation is associated with impaired neuroinflammatory resolution.	Bazinet 2014 [167]; Montine 2002 [168]; Wang 2015 [169]; Serhan 2017 [10]
Polycystic Ovary Syndrome (PCOS)	Insulin resistance, hyperandrogenism, chronic low-grade inflammation	*n*-3 PUFA supplementation improves insulin resistance and inflammatory markers; chronic low-grade inflammation contributes to ovarian dysfunction.	Albardan 2024 [170]; González 2012 [171]
Sarcopenic Obesity (SO)/Metabolic Muscle Disease (MMD)	Mitochondrial overload, impaired fatty acid oxidation, intramyocellular lipid accumulation	Lipotoxic intermediates such as DAG and ceramides impair insulin signaling; PUFA patterns modulate inflammatory tone and may influence the lipotoxic signature in skeletal muscle.	Samuel 2012 [88]; Ertunc 2016 [162]
Cardio-Renal-Metabolic Syndrome (CRMS)/MASLD-related CKD	Systemic inflammation, endothelial dysfunction, metabolic inflexibility	Shared lipotoxic and inflammatory pathways link heart, kidney, liver, and vasculature. More favorable HUFA profiles and higher *n*-3 PUFA intake have been associated with improved cardiometabolic status and lower CKM-related risk markers in population studies.	Lands 1992 [159]; Lands 2008 [58]; Săndulescu 2025 [177]; Zhang 2026 [178]

Abbreviations: HFpEF-like phenotypes = heart failure with preserved ejection fraction–like phenotypes describe early or subclinical metabolic-inflammatory cardiac alterations (e.g., diastolic dysfunction, endothelial dysfunction, myocardial stiffness) that mirror the pathophysiology of HFpEF but occur in the absence of overt, clinically diagnosed HFpEF; CKM severity = Cardiovascular-Kidney-Metabolic (CKM) classification of disease severity.

## Data Availability

No new data were created or analyzed in this study.
